# Structure and vascular function of MEKK3–cerebral cavernous malformations 2 complex

**DOI:** 10.1038/ncomms8937

**Published:** 2015-08-03

**Authors:** Oriana S. Fisher, Hanqiang Deng, Dou Liu, Ya Zhang, Rong Wei, Yong Deng, Fan Zhang, Angeliki Louvi, Benjamin E. Turk, Titus J. Boggon, Bing Su

**Affiliations:** 1 Department of Pharmacology, Yale University School of Medicine, 333 Cedar Street, New Haven, Connecticut 06520, USA; 2 Department of Microbiology and Immunology, Shanghai Institute of Immunology, Shanghai Jiao Tong University School of Medicine (SJTU-SM), Shanghai 200025, China; 3 Department of Immunobiology and the Vascular Biology and Therapeutics Program, Yale University School of Medicine, 333 Cedar Street, New Haven, Connecticut 06520, USA; 4 Departments of Hematology and Dermotology, XiangYa Hospital, Central South University, Changsha 410008, China; 5 Department of Neurosurgery, Yale University School of Medicine, 333 Cedar Street, New Haven, Connecticut 06520, USA

## Abstract

Cerebral cavernous malformations 2 (CCM2) loss is associated with the familial form of CCM disease. The protein kinase MEKK3 (MAP3K3) is essential for embryonic angiogenesis in mice and interacts physically with CCM2, but how this interaction is mediated and its relevance to cerebral vasculature are unknown. Here we report that *Mekk3* plays an intrinsic role in embryonic vascular development. Inducible endothelial *Mekk3* knockout in neonatal mice is lethal due to multiple intracranial haemorrhages and brain blood vessels leakage. We discover direct interaction between CCM2 harmonin homology domain (HHD) and the N terminus of MEKK3, and determine a 2.35 Å cocrystal structure. We find *Mekk3* deficiency impairs neurovascular integrity, which is partially dependent on Rho–ROCK signalling, and that disruption of MEKK3:CCM2 interaction leads to similar neurovascular leakage. We conclude that CCM2:MEKK3-mediated regulation of Rho signalling is required for maintenance of neurovascular integrity, unravelling a mechanism by which *CCM2* loss leads to disease.

## Abstract (web summary)

The protein kinase MEKK3 interacts with CCM2, which is associated with the predominantly cerebrovascular CCM disease. Here the authors use structural, biochemical, cell biology and *in vivo* techniques to show that regulation of Rho signalling by the CCM2:MEKK3 complex is needed to maintain neurovascular integrity.

## Graphical abstract

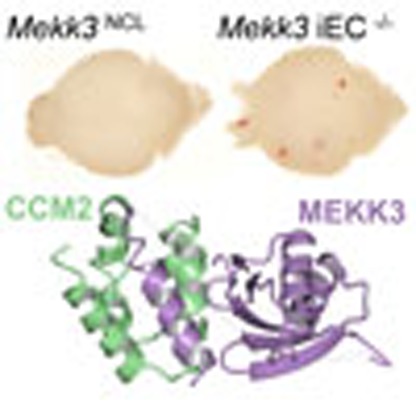

Cerebral cavernous malformations 2 (CCM2) loss is associated with the familial form of CCM disease, manifesting as cerebrovascular lesions that can lead to focal neurological defects and stroke^1^,^2^,^3^. The aetiology and cellular and molecular mechanisms of CCM disease remain elusive. MEKK3 (MAP3K3) is a highly conserved protein kinase essential for embryonic angiogenesis in mice^4^. MEKK3 interacts physically with CCM2 (refs 5, 6) but how this interaction is mediated, and its relevance to cerebral vasculature, are not known.

Here we show that *Mekk3* plays an endothelial cell intrinsic role in embryonic vascular development. Inducible endothelial knockout (*iEC*^*−/−*^) of *Mekk3* in neonatal mice is lethal due to multiple intracranial haemorrhages and massive leakage of brain blood vessels. We discover that MEKK3 is required to suppress a Rho–ROCK-mediated myosin light-chain phosphorylation, a pathway implicated in CCM pathology. We find a direct interaction between the harmonin homology domain (HHD) of CCM2 and the N-terminal region of MEKK3 encompassing both an N-terminal helix and the Phox/Bem1p (PB1) domain, and determine a 2.35-Å cocrystal structure of the complex. We further find that *Mekk3* deficiency impairs neurovascular integrity and that this is partially dependent on the Rho–ROCK signalling. Structure-directed disruption of the MEKK3:CCM2 interaction with a competitive cell-permeable peptide leads to neurovascular leakage that resembles that of MEKK3-deficient endothelial cells. We conclude that regulation of Rho signalling by the CCM2:MEKK3 complex is required for maintenance of neurovascular integrity *in vivo*, unravelling a mechanism by which *CCM2* loss leads to disease.

## Results

### MEKK3 is essential for embryonic vascular development

Germline knockout (KO) of *Mekk3* in mice causes embryonic lethality due to defective blood vessel development^4^,^7^. To examine the role of *Mekk3* specifically in endothelial cells (EC), we generated EC-specific *Mekk3* conditional KO mice (*Mekk3* EC–cKO) by crossing the *Mekk3*^fl/fl^ mice to Tie2-Cre mice. EC-specific conditional inactivation of *Mekk3* leads to lethality around embryonic day (E) 11. At E8.5, *Mekk3* EC–cKO embryos and yolk sacs were indistinguishable from the normal control littermates (NCL; Supplementary Fig. 1a). At E9.5, *Mekk3* EC–cKO embryos were similar in size to their NCL embryos, and whole-mount anti-CD31 staining revealed severely impaired angiogenesis in *Mekk3* EC–cKO embryos (Supplementary Fig. 1b,c).

**Figure 1:**
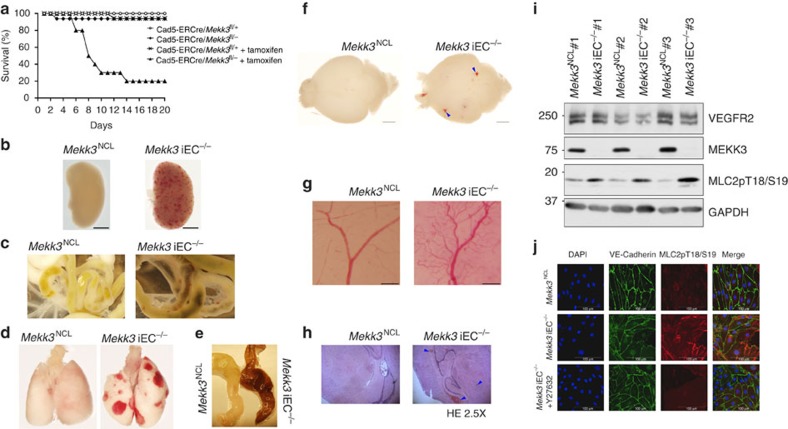
MEKK3 is essential for embryonic vascular development and neonatal brain vascular integrity. (**a**) Survival lines of neonatal pups of normal control littermate (NCL) Cad5-ERCre/*Mekk3*^*fl/+*^ (open circles, *n*=17) or Cad5-ERCre/*Mekk3*^*fl/−*^ (black diamonds, *n*=16) with no tamoxifen treatment or treated with 5 μl of tamoxifen (10 mg ml^*−*1^) orally at day-1 after birth and continuously daily (Cad5-ERCre/*Mekk3*^*fl/+*^, black crosses, *n*=17; Cad5-ERCre/*Mekk3*^*fl/−*^, black triangles, *n*=10). The pups were observed for 20 days for survival. (**b**–**g**) Organ examples collected from neonatal littermates of tamoxifen-treated Cad5-ERCre/*Mekk3*^*fl/+*^ (*Mekk3*^NCL^) pups or EC-specific *Mekk3* deletion Cad5-ERCre/*Mekk3*^*fl/−*^ (*Mekk3* iEC^*−/−*^) pups as indicated: (**b**) kidneys of postnatal day (P)10 littermate pups, (**c**) small intestines from P10 littermate pups, (**d**) lungs from P6 littermate pups, (**e**) colons from P10 littermate pups, (**f**) brains from P7 littermate pups. (**g**) Light microscopic pictures showing brain surface of P7 littermate pups. Scale bar, 5 mm. Arrowheads in **f** show bleeding spots observed from the surface of *Mekk3* iEC^*−/−*^ brain. (**h**) Haematoxylin and eosin staining of paraffin-embedded sections of P7 neonatal *Mekk3*^NCL^ and *Mekk3* iEC^*−/−*^ brains visualized by a light microscope under a 2.5 × objective lens. Blue arrowheads indicate the haemorrhage sites inside the brains. (**i**) Immunoblots show expression of VEGFR2, MEKK3 and T18/S19 phosphorylated MLC2 (MLC2pT18/S19) in freshly isolated BMEC from three *Mekk3*^NCL^ and three *Mekk3* iEC^*−/−*^ littermate pups as indicated. GAPDH expression is shown as loading controls. Elevated MLC2pT18/S19 was observed in three independently isolated pairs. (**j**) Confocal microscopy to visualize the expression of VE-Cadherin (green) and MLC2pT18/S19 (red) in primary BMEC from P7 *Mekk3*^NCL^, or *Mekk3* iEC^*−/−*^, or *Mekk3* iEC^*−/−*^ pups treated with Y27632. Cell nuclei were stained with DAPI (blue). MLC2pT18/S19 was elevated in *Mekk3* iEC^*−/−*^BMEC, which was suppressed by addition of ROCK inhibitor Y27632. Scale bar, 100 μm.

To understand the impact of *Mekk3* loss at a later stage of development and its role in normal physiological and pathological settings, we generated EC-specific tamoxifen-inducible *Mekk3* iKO mice (*Mekk3 iEC*^*−/−*^). *Mekk3* deletion was induced at postnatal day (P) 1 by oral feeding of tamoxifen daily. *Mekk3 iEC*^*−/−*^ pups became obviously sick 2–3 days after the initiation of tamoxifen administration, and most died within 15 days (Fig. 1a). In contrast, control *Mekk3 iEC*^*+/−*^ pups, although only having one copy of the *Mekk3* gene in EC, survived at similar doses of tamoxifen (Fig. 1a), as did the untreated pups (Fig. 1a). The *Mekk3 iEC*^*−/−*^ pups showed haemorrhage in various organs, including the kidney, small intestine, lung and colon (Fig. 1b–e). Although neonatal loss of MEKK3 could result in haemorrhages of multiple organs, the brain is the most affected organ, which is also likely the main cause of lethality (Fig. 1). In fact, almost all the *Mekk3 iEC*^*−/−*^ pups showed brain haemorrhage, but only a fraction of other organs were affected. This may be due to fact that brain vascular MEKK3 is more efficiently deleted by tamoxifen administration. Interestingly, we observed that about 50% of *Mekk3 iEC*^*−/−*^ pups developed signs of paralysis before death. Indeed, after 5–7 days of tamoxifen treatment, the *Mekk3 iEC*^*−/−*^ pups harboured multiple focal haemorrhages on the surface and inside of the brain, but this was not observed in control brains (*Mekk3 iEC*^*+/−*^ treated with tamoxifen termed *Mekk3*^NCL^; Fig. 1f). The neonatal vasculature was also malformed when *Mekk3* was deleted (Fig. 1g, Supplementary Fig. 2) and haematoxylin and eosin staining of the neonatal brain confirmed bleeding in the cerebella and brain surface areas (Fig. 1h, Supplementary Fig. 3). The postnatal loss of *Mekk3* in the endothelium is therefore associated with severe haemorrhage in the brain vasculature.

The above data indicate a critical role of MEKK3 in the neurovascular function. Interestingly, the neurovascular defects in *Mekk3 iEC*^*−/−*^ pups are reminiscent of those of *Ccm2* KO mice suggesting a genetic link of these two genes in the brain vascular function^8^,^9^,^10^. Although the MEKK3–CCM2 interaction may play a role in stabilizing CCM2, we did not observe any obvious decrease of CCM2 in the MEKK3 KO cells suggesting that MEKK3 may not regulate the stability of CCM2. Rho and ROCK signalling have, however, been implicated as downstream targets of CCM2 (refs 10, 11), and previous work showed that haploinsufficiency of either *Krit1* or *Ccm2* increases *in vivo* vascular leakage, that MLC phosphorylation increased in patient samples, and that in haploinsufficient cells a ROCK inhibitor was able to restore barrier function^11^. Therefore, we wondered whether MEKK3 also participates in mediating Rho/ROCK activity in the developing brain vasculature. To test whether there were changes in Rho/ROCK signalling in the *Mekk3 iEC*^*−/−*^ mice we probed for phosphorylation of the downstream substrate of Rho/ROCK, Myosin Light Chain 2 (MLC2). We found that on loss of MEKK3 expression, MLC2 phosphorylation increased markedly (Fig. 1i,j). Consistently we also found that treatment with a ROCK inhibitor (Y27632) reduces the phosphorylation of MLC2 (Fig. 1j) confirming that MLC2 phosphorylation is occurring through ROCK activity.

### MEKK3 NPB1 region interacts with the HHD domain of CCM2

**Figure 2:**
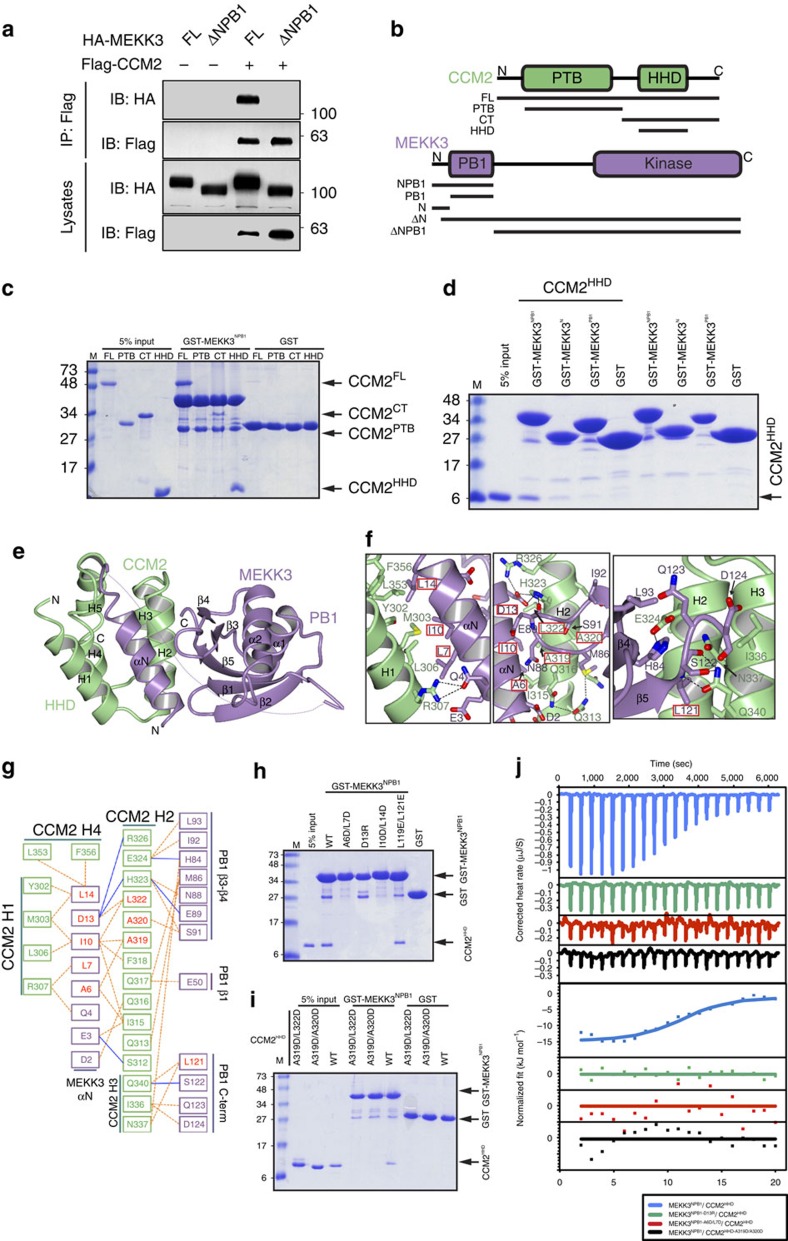
MEKK3 uses its NPB1 region to interact with the HHD domain of CCM2. (**a**) Immunoprecipitation (IP) of Flag–CCM2 after cotransfection with HA–MEKK3^FL^ or HA–MEKK3^ΔNPB1^ into 293T cells. Whole-cell lysates and immunoprecipitates were analysed by immunoblotting (IB) with the indicated antibodies. (**b**) Domain schematic of CCM2 (top) and MEKK3 (bottom). Black lines indicate constructs used in this study. Domains are indicated as PTB (Phosphotyrosine Binding), HHD (Harmonin homology domain), PB1 (Phox/Bem1p) and kinase. N and C termini are indicated. (**c**) Pull-down assay. GST–MEKK3^NPB1^ was immobilized on glutathione Sepharose beads and tested for ability to pull-down purified regions of CCM2. Coomassie stained SDS–PAGE analysis of washed beads. (**d**) Pull-down assay. Immobilized GST–MEKK3^NPB1^, GST–MEKK3^N^ or GST–MEKK3^PB1^ were incubated with purified CCM2^HHD^. Coomassie-stained SDS–PAGE of washed beads. (**e**) Cocrystal structure of MEKK3^NPB1^ in complex with CCM2^HHD^. CCM2^HHD^ is shown in green and MEKK3^NPB1^ in purple. Secondary structure elements are labelled. Disordered residues indicated by a dotted line. N and C termini are indicated. (**f**) Close-up views of the main interaction sites between MEKK3^NPB1^ and CCM2^HHD^. Left, CCM2 helix H1 with MEKK3 helix αN. Centre, CCM2 helix H2 with MEKK3 helix αN and PB1 domain. Right, CCM2 helices H2 and H3 with MEKK3 PB1 domain. Hydrogen-bonds shown as dotted lines. Residues mutated in this study are boxed in red. Structural figures generated using CCP4mg^36^. (**g**) Map of MEKK3^NPB1^:CCM2^HHD^ interactions. MEKK3 residues are shown in purple and CCM2 residues in green. Residues mutated in this study are shown in red. Dotted lines indicate hydrophobic interactions and solid lines ionic interactions. Interactions defined by PDBSum^37^. (**h**) Pull-down assay. Crystallographically determined mutations to GST–MEKK3^NPB1^ interrupts pull-down of wild-type CCM2^HHD^. MEKK3 mutants, A6D/L7D, D13R, I10D/L14D and L119E/L121E are indicated. Coomassie stained SDS–PAGE of washed beads. (**i**) Pull-down assay. Introduction of crystallographically determined mutations to CCM2^HHD^ interrupts pull-down with wild-type GST–MEKK3^NPB1^. CCM2 mutants A319D/L322D and A319D/A320D are indicated. Coomassie stained SDS–PAGE of washed beads. (**j**) Isothermal titration calorimetry. CCM2^HHD^ was titrated into MEKK3^NPB1^. Crystallographically defined mutants tested for MEKK3 were D13R and A6D/L7D and for CCM2 was A319D/A320D^38^.

As the *Mekk3 iEC*^*−/−*^ data above support a genetic link between *Mekk3* and *Ccm2*, we next asked whether these neurovascular phenotypes directly relate to an interaction between MEKK3 and CCM2. As previously reported^5^, we found that full-length MEKK3 and full-length CCM2 co-immunoprecipitate (Fig. 2a). MEKK3 contains only two defined domains: an N-terminal Phox/Bem1p (PB1) domain and a C-terminal catalytic domain. Because MEKK3’s PB1 domain is a known protein interaction domain we tested whether MEKK3 lacking this region (MEKK3^ΔNPB1^) could bind CCM2 and found that the interaction was lost, indicating that the PB1 domain was important for MEKK3–CCM2 interaction (Fig. 2a). To probe this interaction more deeply we generated a number of constructs of CCM2 and MEKK3 and examined direct binding *in vitro* (Fig. 2b). We found a direct interaction between the N terminus of MEKK3 (MEKK3^NPB1^) and full-length CCM2 (CCM2^FL^) using purified components (Fig. 2c). We further tested constructs of CCM2 encoding its phosphotyrosine binding (PTB) domain^12^ (CCM2^PTB^), its C terminus (CCM2^CT^), and its HHD (CCM2^HHD^)^13^ (Fig. 2b) and found that CCM2^HHD^ directly interacts with GST–MEKK3^NPB1^, but that CCM2^PTB^ does not (Fig. 2c). This represents the first binding partner identified for the CCM2 HHD. Further pull-down analysis showed that neither the PB1 domain (GST–MEKK3^PB1^) nor the predicted α-helical N terminus of MEKK3 (GST–MEKK3^N^) could appreciably pull-down CCM2^HHD^ on their own, but that a construct encoding both of these regions of MEKK3 (GST–MEKK3^NPB1^) could (Fig. 2d). This suggested that both the canonical protein interaction PB1 domain of MEKK3 and a short region N-terminal of this domain play roles in its interaction with CCM2.

To better understand how CCM2 and MEKK3 interact we determined a cocrystal structure of MEKK3^NPB1^ with CCM2^HHD^ to 2.35 Å resolution (Table 1; Fig. 2e). In addition to the HHD and PB1 domains, we observed a stretch of positive difference density between CCM2^HHD^ helices H1 and H2. This difference density corresponded to an additional helix, which, after further rounds of refinement, we identified and built as the N-terminal 19 residues of MEKK3 (Supplementary Fig. 4a,b). Although this region of MEKK3 is well-conserved over evolution (Supplementary Fig. 5) it has never before been proposed to mediate binding to protein interaction partners, and its extensive interaction with CCM2 was unexpected.

The overall structure of the CCM2^HHD^:MEKK3^NPB1^ complex reveals an extensive interface between the two proteins. MEKK3 resembles a hand, with the N-terminal helix forming the thumb and the PB1 domain forming the palm/fingers. Like other PB1 domains, the MEKK3^PB1^ adopts a ubiquitin-like fold consisting of a five-stranded β-sheet, an α-helix inserted between strands β2 and β3, and a second helix between β4 and β5 (ref. 14). MEKK3 grasps the CCM2^HHD^, with the thumb (MEKK3^N^) resting in the groove between CCM2 helices H1 and H2, and the palm/fingers (MEKK3^PB1^) holding CCM2 helices H2 and H3 (Fig. 2e). The N-terminal helix (αN) of MEKK3 fits snugly into the hydrophobic groove between CCM2 helices H1/H4 and H2, making extensive interactions with them (Fig. 2e,f,g, Supplementary Fig. 4c). On the basis of the crystal structure we introduced point mutations into CCM2^HHD^ and MEKK3^NPB1^ to interrupt the interaction by pull down (Fig. 2h,i). We found that mutants in MEKK3 helix αN (A6D/L7D, D13R, I10D/L14D) severely interrupted CCM2 pull down (Fig. 2h), but mutation within the MEKK3 PB1 domain (L119E/L121E) only moderately reduced CCM2^HHD^ pull down (Fig. 2h). Mutations in CCM2^HHD^ also interrupt its ability to pull down with MEKK3 (Fig. 2i). Isothermal titration calorimetry showed an observed *K*_d_ of 1.4±0.4 μM (Fig. 2j, Supplementary Table 1) for the wild-type (WT) components, but no heat of interaction was observed for CCM2^HHD^ (A319D/A320D), MEKK3^NPB1^ (A6D/L7D) or MEKK3^NPB1^ (D13R) mutants tested (Fig. 2j).

### Role of MEKK3:CCM2 interaction in signalling and localization

**Figure 3:**
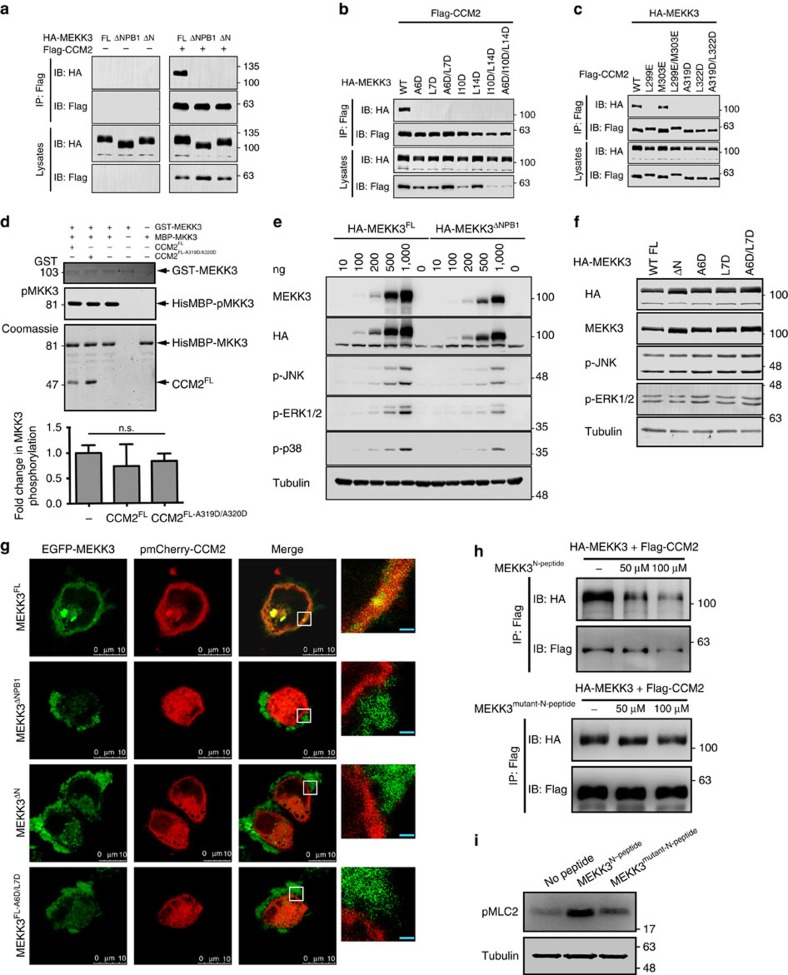
Role of MEKK3:CCM2 interaction in signaling and localization. (**a**) Flag-tagged full-length CCM2 (Flag–CCM2) was cotransfected with HA–MEKK3^FL^, HA–MEKK3^ΔNPB1^ or HA–MEKK3^ΔN^ into 293T cells as indicated. Transfected cells were subjected to immunoprecipitation (IP) and the immunoprecipitates and the whole-cell lysates were further analysed by IB with the indicated antibodies. (**b**) HA-tagged wild-type MEKK3 (WT) or its derived mutants A6D, L7D, A6D/L7D, I10D, L14D, I10D/L14D and A6D/I10D/L14D were cotransfected and analysed for interaction with Flag–CCM2 by co-IP. (**c**) HA–MEKK3 was cotransfected with Flag–CCM2, or its derived mutants L299E, M303E, L299E/M303E, A319D, L322D and A319D/L322D. The MEKK3–CCM2 interaction was then analysed by co-IP. (**d**) MBP–MKK3 was phosphorylated by GST–MEKK3 in the absence or presence of purified WT full-length CCM2 (CCM2^FL^) or its derived mutant (CCM2^FL-A319D/A320D^) as indicated. Loading of GST–MEKK3, HisMBP–MKK3, and CCM2 shown in the top and bottom panels, respectively. Phosphorylated HisMBP-MKK3 shown in the middle panel. Quantification was performed using a LiCor Odyssey Imager and shown below. Error bars indicate s.d. *N*=3. (**e**) 293T cells were transfected with HA–MEKK3^FL^ or HA–MEKK3^ΔNPB1^. Cell extracts were prepared and analysed for p-JNK, p-ERK1/2 and p-p38 levels by IB with the indicated antibodies. Relative HA–MEKK3 expression levels shown by IB with an anti-HA antibody. (**f**) 293T cells were transfected with HA–MEKK3^FL^, HA–MEKK3^ΔN^, HA–MEKK3^FL-A6D^, HA–MEKK3^FL-L7D^ or HA–MEKK3^FL-A6D/L7D^. Cell extracts were analysed for p-JNK and p-ERK1/2 levels by IB. Relative expression levels of MEKK3 and its mutants were determined by IB with an anti-HA antibody. (**g**) Representative confocal images showing that CCM2 colocalizes with WT MEKK3 (MEKK3^FL^), but not its mutants MEKK3^ΔNPB1^, MEKK3^ΔN^ or MEKK3^FL-A6D/L7D^. Visualized with a LM8 Leica confocal microscopy. White scale bar, 10 μm; cyan scale bar, 1 μm. Power of objective lens: 63 × . (**h**) MEKK3^N-peptide^ disrupts CCM2–MEKK3 interaction. Flag–CCM2 and HA–MEKK3 were cotransfected into 293T cells. After 24 h, the transfected cells were administrated with cell-permeable peptides MEKK3^N-peptide^ or MEKK3^mutant-N-peptide^. Flag–CCM2 and HA–MEKK3 interaction was determined by co-IP. (**i**) MEKK3^N-peptide^ increases pMLC2 S19 phosphorylation. Mouse brain endothelial cells from WT pups were isolated, cultured for 3 days and treated with either MEKK3^N-peptide^ or MEKK3^mutant-N-peptide^.

We found that in accordance with the *in vitro* experiments, introduction of the structure-designed mutations into the full-length proteins disrupted their interaction in cultured cells as measured by coimmunoprecipitation (Fig. 3a–c). As MEKK3 is a protein kinase we next wondered whether the interaction with CCM2 would impact kinase activity. We found that CCM2 had no effect on MEKK3 activity toward a purified substrate (MKK3) *in vitro* (Fig. 3d). Likewise, the interaction with CCM2 did not alter MEKK3 signalling to the ERK1/2, p38 or JNK MAPK cascades in cells (Fig. 3e,f) suggesting an alternate role for CCM2 interaction with MEKK3 in vascular function. Fluorescently tagged full-length MEKK3 and CCM2 colocalized in the para-plasma membrane region in HeLa cells (Fig. 3g). In contrast, coexpression of MEKK3 lacking either the NPB1 region or the N-terminal helix alone (MEKK3^ΔNPB1^, MEKK3^ΔN^) did not colocalize with CCM2. MEKK3 remained predominantly at the membrane, but CCM2 appeared to be diffused throughout the cytosol rather than close to the membrane (Fig. 3g). Likewise, coexpression of CCM2 with MEKK3 that included a double mutation (A6D/L7D), which prevents interaction with CCM2 (MEKK3^FL-A6D/L7D^) failed to show colocalization. We therefore conclude that the interaction between CCM2 and MEKK3 is important for correct subcellular localization of CCM2. It is also formally possible that CCM2 is required for activation of a spatially restricted pool of MEKK3.

As all of the above data suggest the importance of the N-terminal region of MEKK3 for its interaction with CCM2 we predicted that a peptide based on the most N-terminal region of MEKK3 would directly compete with MEKK3 for binding to CCM2 (Fig. 2e). To test this we designed a cell-permeable peptide based on the N-terminal portion of MEKK3 encompassing residues 2–18 of MEKK3 (MEKK3^N-peptide^) that we predicted would interrupt the MEKK3–CCM2 interaction. We also designed an identical peptide that included a double mutation A6D/L7D that prevents interaction with CCM2 (MEKK3^mutant-N-peptide^). We found that MEKK3^N-peptide^ disrupts coimmunoprecipitation with CCM2 (Fig. 3h) but MEKK3^mutant-N-peptide^ does not (Fig. 3h), and that MEKK3^N-peptide^ increases MLC2 phosphorylation in isolated WT mouse brain endothelial cells, an effect that was reduced on treatment with MEKK3^mutant-N-peptide^ (Fig. 3i). As expected, we found that the MEKK3^N-peptide^ also colocalizes with CCM2 in transfected cells suggesting that it interacted CCM2 in cells (Supplementary Fig. 6).

### MEKK3 regulates the neonatal vascular permeability

**Figure 4:**
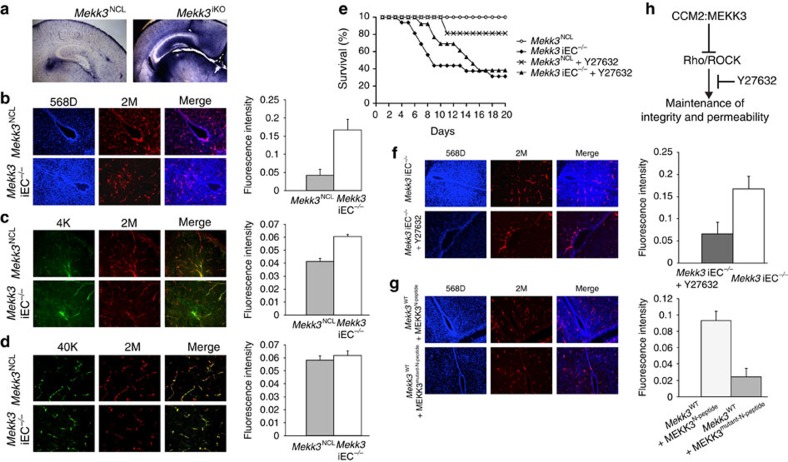
MEKK3 critically regulates the neonatal vascular permeability to small size molecule via suppressing the Rho signals. (**a**–**d**) *In vivo* leakage experiments show only small-molecular-weight tracers leaked from brain microvessels in Mekk3 iKO and iEC^*−/−*^ neonatal mice. (**a**) Sulfo-NHS-biotin (556.6 dalton) was injected into the hearts of the tamoxifen-treated *Mekk3* NCL or *Mekk3* iKO neonatal pups. Brain sections were analysed for Sulfo-NHS-biotin leakage by staining. Objective lens power: 2.5 × . (**b**–**d**) Fluorescent-labelled tracers with different molecular weights were injected into tamoxifen-treated *Mekk3* NCL or *Mekk3* iEC^*−/−*^ P7 neonatal pups’ hearts. After euthanization, brains were fixed and sectioned and tracer leakage to the brain was determined. (**b**) Hoechst 33342 (616 dalton; blue) plus Dextran–Rhodamine (2 M Dalton; red), *N*=7, (**c**) Dextran–FITC (MW: 4K Dalton; green) plus Dextran–Rhodamine (2 M Dalton)(red), N=4, (**d**) Dextran–FITC (40 K Dalton; green) plus Dextran–Rhodamine (2 M Dalton; red). Bar graphs on the left of each panel show quantification of the relative leakage of the tracers normalized to the intensity of Dextran–Rhodamine, and shows no leakage in either NCL nor *Mekk3* iEC^*−/−*^ neonatal pups. Error bars indicate s.d. *N*=4. Objective lens power: 10 × . (**e**) ROCK inhibitor Y27632 partially rescues survival of *Mekk3* iEC^*−/−*^ neonatal pups. NCL or *Mekk3* iEC^*−/−*^ pups were treated with tamoxifen at P1, and continued daily in the absence (open circle and black diamond, respectively) or presence of Y27632 (cross and black triangle, respectively). Survival of pups was monitored daily until P20. (**f**) Hoechst 33342 (616 Dalton; blue) plus Dextran K–Rhodamine (2 M Dalton; red) were injected into tamoxifen-treated *Mekk3* iEC^*−/−*^ P7 neonatal pups’ hearts fed either with water or Y27632. After euthanization, brains were fixed and sectioned at 30-μm thickness and leakage determined as described above. *N*=5. Objective lens power: 10 × . (**g**) Wild-type P7 neonatal pups were treated with cell-permeable wild-type *Mekk3*-peptide (MEKK3^N-peptide^) or A6D/L7D mutated *Mekk3*-peptide (MEKK3^mutant-N-peptide^). Brain leakage determined using Hoechst 33342 (blue) plus Dextran K–Rhodamine (red). *N*=5. Objective lens power: 10 × . (**h**) Proposed signalling pathway. The interactions of CCM2 and MEKK3 are critical for maintenance of vasculature integrity and permeability by control of Rho/ROCK signalling.

The above biochemical, biophysical and *in vitro* studies provided the first direct evidence to link the physiological function of an interaction between MEKK3 and CCM2 in brain vasculature. Since ample evidence has suggested the importance of CCM proteins in endothelial cell junctions^15^,^16^,^17^,^18^, to better understand the impact of *Mekk3* deletion in brain vasculature development, we tested if there were any EC cell permeability alterations in the neurovasculature of developing mice. To understand why MEKK3 deficiency causes brain haemorrhage, we examined the integrity of brain blood vessels in *Mekk3 iEC*^*−/−*^ and control mice. We used Sulfo-NHS-biotin to visualize the brain vessels and detect possible leaks. Normal control littermate pups or *Mekk3* iKO pups were fed tamoxifen from day P1 and analysed at P6, a time-point chosen because it is before the onset of massive cerebral haemorrhage. In contrast to the control brains, the *Mekk3* iKO brains showed massive leakage of Sulfo-NHS-biotinylated materials, which stained blue (Fig. 4a). The leakage appeared throughout much of the brain in the cerebral cortex, thalamus and hypothalamus areas indicating a defective brain blood barrier. The hippocampus area of the *Mekk3* iKO brains showed significant blue staining compared to the control hippocampus, strongly indicating that the defect was due to blood vessel leakage.

Because brain endothelial junctions have multiple layers of regulation for different sized molecules to permeate the junction, we tested permeability of different sized dyes for the *Mekk3 iEC*^*−/−*^ mice (Fig. 4b–d). We found that although the vasculature was intact for 2 M, 40 K and largely for 4 K dalton dyes, there was extensive leakage for the 568 dalton dye. This result shows that MEKK3 function in brain endothelial cells is important for control of cellular permeability to a relatively small-molecular-weight material but may be not required for permeability of large-molecular-weight materials. As we had found that a ROCK inhibitor (Y27632) could reduce MLC2 phosphorylation in *Mekk3 iEC*^*−/−*^ cells (Fig. 1j) we wondered whether it could rescue the survival of *Mekk3 iEC*^*−/−*^ neonatal mice. We found that although there is a slight toxicity to the WT pups at the dose we used, administration of the same dose rescued the median survival of *Mekk3 iEC*^*−/−*^ mice (day 9 for *Mekk3 iEC*^*−/−*^ and day 15 for *Mekk3 iEC*^*−/−*^+Y27632). These data strongly suggest that MEKK3 may participate in CCM-mediated Rho/ROCK suppression in normal brain vasculature (Fig. 4e, Supplementary Tables 2,3). To further show that Rho/ROCK activity associated with MEKK3 loss plays a role in inducing neonatal brain vasculature leakage, we tested the ROCK inhibitor in *Mekk3 iEC*^*−/−*^ neonatal pups to see if the leaky phenotype could be rescued. Indeed, we find that administration of the inhibitor results in significantly less brain vasculature leakage as compared with untreated *Mekk3 iEC*^*−/−*^ mice (Fig. 4f).

To show that the MEKK3:CCM2 interaction is essential for neonatal brain vasculature function we took advantage of the cell-permeable peptide that we had shown to be able to disrupt binding (Fig. 3g, Supplementary Figs 6,7,8). We predicted that if the specific MEKK3:CCM2 interaction was in fact critical for protecting the brain vasculature from small-molecular-weight molecule permeability, then infusion of this peptide to neonatal WT mice should lead to increased brain vascular permeability to small-molecular-weight substances, but not large-molecular-weight material. Indeed, we find that mice that were given the cell-permeable competitive peptide (MEKK3^N-peptide^) showed significant brain vasculature permeability to small-molecular-weight but not large-molecular-weight dyes (Fig. 4g). For comparison, the control WT mice were treated with a control mutant peptide (MEKK3^mutant-N-peptide^) that we showed does not interrupt CCM2:MEKK3 interaction (Fig. 3d). We found that the control mutant peptide does not impact brain vasculature permeability (Fig. 4g).

## Discussion

Although both MEKK3 and CCM2 signalling have been known for over a decade to play roles in regulation of development and of vasculature, no previous studies have linked their similar vasculature functions by mouse models or at the structural level. The current study is the first to bring these two major vasculature regulating proteins together to show their essential role in regulating Rho-/ROCK-dependent brain endothelial permeability to small-molecular-weight substances. These data also suggest that permeability due to loss of the MEKK3:CCM2 interaction is likely the underlying reason for defects in the developing vasculature.

The precise mechanism of CCM complex-mediated regulation of Rho signalling remains undetermined. Most studies in the CCM field have reached the consensus that this regulation is related to normal brain vasculature function and that deregulated Rho/ROCK signalling is associated with acquisition of CCM lesions^17^,^19^. Our current study has narrowed the gap for understanding this regulation mechanism (Fig. 4h). At the moment, we do not understand how MEKK3 may suppress the Rho/ROCK signalling in EC, which could occur at multiple levels along the vascular growth factor-mediated signalling pathways. In this regard, we found that in MEKK3 KO ECs the protein level of RhoA appeared upregulated, providing one possible mechanism for such a regulation of Rho/ROCK signals by CCM2:MEKK3. It would be fruitful to for further studies unravel how CCM2:MEKK3 interaction is involved in suppression of Rho/ROCK signalling to maintain vascular integrity.

## Methods

### Mice and *in vivo* experiments

*Mekk3*^+/*−*^ and *Mekk3*^fl/fl^ mice were generated from our laboratory^4^,^20^. Tie2-Cre mice were obtained from Richard Flavell’s lab^21^. ROSA26R mice were developed in Philippe Soriano’s group^22^; ROSA26-ERCre mice were generated in Andrew McMahon’s lab^23^; ROSA26GFP mice were from Frank Costantini’s group^24^; and Cdh5-CreERT2 mice were obtained from Dr Wang Min’s group at Yale University originally described by Ralf Adams^25^. Methods for the experiments with yolk sacs and embryos were described in our previous study by Yang *et al*.^4^. Breeding of the above mouse lines to generate appropriate combinations of experimental mice and the mouse experiments are in compliance with ethical regulations and are approved by the Institutional Animal Care and Use Committee (IACUC) of Yale University (Approve animal protocol number: IACUC # 2012-11094).

Tamoxifen (dissolved in sterile corn oil at a concentration of 10 mg ml^*−*1^) was administered to neonatal MEKK3^fl/+;Cdh5CreERT2^ and MEKK3^fl/*−*;Cdh5-CreERT2^ pups by feeding to the mouth starting at postnatal day 0–1 (P0–1). The tamoxifen feeding schedule was: P0–2, 5 μg per day; P3–5, 7.5 μg per day; after P6, 10 μg per day. The P7 pups were used for brain leakage study with fluorescent-labelled tracers via intracardiac injection, or continually fed and monitored until P21 for the survival experiments. Specifically, the P7 pups were first anaesthetized with ketamine (100 mg kg^*−*1^) and Xylamine (10 mg kg^*−*1^) administered by a single intraperitonial (i.p.) injection. Either of the following solutions were injected into the left cardiac ventricle of the pups through the diaphragm of the anaesthetized pups within 1 min under a microscope^26^: (1) 50 μl of fluorescent-labelled tracer: mix 1:1 of Hoechst 33342 (Cat#: H3760, Invitrogen, 10 mg ml^*−*1^ solution in water) and Dextran conjugated with either Rhodamine or FITC (10 mg ml^*−*1^ in water); (2) 50 μl of Sulfo-NHS-biotin: freshly prepared cell surface biotinylation solution (EZ-link Sulfo-NHS-biotin (Pierce #21217) 0.5 mg g^*−*1^ body weight diluted in PBS per 1 mM CaCl_2_). Three minutes after injection, the pups were euthanized with CO_2_. Brains would then be removed and fixed with 3.7% formaldehyde, then sectioned and analysed. Quantification of fluorescence intensity from each molecular weight dye is measured with Image-J software and the bar graphs on the left of each panels show the relative leakage of the Hoechst, 4 K tracer, and 40 K tracer, which was normalized to the intensity of Dextran–Rhodamine (2 million dalton). A minimum of five different pictures were taken randomly per measurement for the average Dextran–Rhodamine intensity using Image-J software, and the coinjected dyes (Hoechst or FITC) on the same area were also measured and normalized to the intensity of the average Dextran–Rhodamine dye. Bar graphs represent the means of three independent measurements with s.d. For the survival experiment, the mice were monitored twice daily. Any pups exhibiting a total of 5% weight loss over the course of two consecutive days or showing signs of impaired limb movement or wobbly gait would be immediately euthanized using CO_2_.

Mouse strain C57/B6, both males and females, of neonatal to 6-month-old mice were used in the study.

### Cell-permeable peptides

The following peptides were synthesized by ChinaPeptides Co., Ltd., Shanghai China

TAT–HA–MEKK3^N-peptide^, GRKKRRQRRRPPQYPYDVPDYADEQEALNSIMNDLVALQ,

TAT–HA–MEKK3^mutant-N-peptide^, GRKKRRQRRRPPQYPYDVPDYADEQEDDNSIMNDLVALQ,

FITC–TAT–MEKK3^N-peptide^, FITC–GGGGGRKKRRQRRRPPQDEQEALNSIMNDLVALQ,

FITC–TAT–MEKK3^mutant-N-peptide^, FITC–GGGGGRKKRRQRRRPPQDEQEDDNSIMNDLVALQ.

### Peptide experiments

TAT–HA–MEKK3 (*Mekk3*^N-peptide^, 2.5 mg ml^*−*1^ in sterile double-distilled water (ddH_2_O)) and TAT–HA–MEKK3–A6D/L7D (*Mekk3*^mutant-N-peptide^, 2.5 mg ml^*−*1^ in sterile ddH_2_O) were administered i.p. to the *Mekk3*^*f*l/fl^ mice starting at postnatal day 0 (P0) according to the following feeding schedule: P1: 10 μl (25 μg) *Mekk3*^N-peptide^ or *Mekk3*^mutant-N-peptide^ per pup; P2-6: 10 μl (25 μg) *Mekk3*^N-peptide^ or *Mekk3*^mutant-N-peptide^ per pup; at P7: brain leakage analysed.

Mouse brain endothelial cells were isolated from WT pups according to the procedures described above. After 3 days, cells were incubated with 50 μM MEKK3^N-peptide^ or MEKK3^mutant-N-peptide^ for 6 h, then were lysed for western blotting with indicated antibodies.

### Rescue experiment

Tamoxifen induction was similarly performed as described above. At the same time, the pups were fed with Y27632 (1 mg ml^*−*1^ in sterile ddH_2_O) from P2 to P14, with a dose of 1 μg g^*−*1^ of bodyweight. Brain leakage was evaluated at P7 as described above, or the survival of the pups was monitored until P21 with continued feeding. The pups were monitored twice daily. Any pups exhibiting a total of 5% weight loss over the course of two consecutive days or showing signs of impaired limb movement or wobbly gait, it would be immediately euthanized.

### Immunohistochemical analysis

The embryos for immunohistochemical and whole-mount staining were fixed in 4% paraformaldehyde. Embryos for immunohistochemical staining were embedded in paraffin and cut into 6- to 7-μm thick sections. Tissues for frozen sections were embedded in OCT (Tissue-Tek) and cut into 8-μm thick sections. The frozen sections were fixed in acetone and acetone–chloroform. The haematoxylin and eosin staining procedure followed standard protocol. The immunohistochemical and immunofluorescent staining with anti-α-smooth muscle actin antibody (1A4, Sigma, 1:400 dilution) or anti-CD31 antibody (MEC13.3, BD, 1:400 dilution) followed basic immunoperoxidase procedures or immunostaining procedure for fluorophore-conjugated secondary antibodies. The procedures for whole-mount anti-CD31 staining were described previously^4^.

### Transient transfection

For transient transfection of *in vitro* cultured cells, Lipofectamine 2000 (Invitrogen) was used according to manufacturer’s instruction. Transfected cells were analysed 24 h later.

### Antibodies

Monoclonal anti-Flag antibody M2 (catalogue number F1804) (dilution 1:1,000) is from Sigma, anti-tubulin antibody was prepared from an anti-tubulin hybridoma clone (12G10) purchased from the Hybridoma Bank at the University of Iowa (1:10,000). Anti-HA antibody was purified from 12CA5 hybridoma^27^ (1:20,000). p-JNK (#4668) (1:1,000), p-ERK1/2 (#9106) (1:2,000), anti-VEGFR2 (#2472) (1:1,000), p-p38 (#9211) (1:1,000), MLC2pT18/S19 (#3674) (1:1,000 for immunoblot, 1:200 for IF), MLC2pS19 (#3671) (1:1,000) anti-mouse IgG and HRP-linked Antibody (#7076) (1:5,000) were purchased from Cell Signaling Technology (MA). Anti-MEKK3 is from R&D (MN) (MAB6095) (1:1,000), and anti-VE-Cadherin is from Santa Cruz Biotechnology (Dallas, TX) (sc-79818) (1:200), anti-α-smooth muscle actin mouse monoclonal antibody (clone 1A4) is from Sigma (A5228) (1:200) and anti-CD31 rat monoclonal antibody (clone MEC13.3) is from BD bioscience (553370) (1:200).

### Dyes and inhibitors

Tamoxifen, ketamine, Xylamine, Dextran–FITC (4 K), Dextran–FITC (40 K) were purchased from Sigma. Hoechst 33342 and Dextran–Rhodamine (2 M) were purchased from Invitrogen. Sulfo-NHS-biotin was freshly prepared using EZ-link Sulfo-NHS-biotin from Pierce #21217. ROCK inhibitor Y27632 was purchased from L.C. Laboratories.

### Construct design

Human *MEKK3* cDNA (UniProt Q99759) was purchased from Open Biosystems (Clone 6183752), and the sequence corresponding to amino acid residues 1–124 was subcloned into a hexahistidine (6xHis)-pCDF vector (Novagen) (modified to include a TEV cleavage site) using EcoRI and SalI restriction sites. The GST-fusion constructs of MEKK3 were produced by subcloning MEKK3 into a GST-pCDF vector (GST–MEKK3^NPB1^: 1–124, GST–MEKK3^N^: 1–36 and GST–MEKK3^PB1^: 37–124). The N-terminally His-tagged CCM2 (UniProt: Q9BSQ5) constructs (CCM2^FL^: 1–438, CCM2^PTB^: 51–251, CCM2^CT^: 231–438, CCM2^HHD^: 283–379) were generated from human cDNA^13^,^28^. Mutations in CCM2^HHD^ (A319D/A320D, A319D/L322D) and MEKK3^NPB1^ (A6D/L7D, D13R, I10D/L14D, and L119D/L121D) were introduced using QuikChange Lightning (Qiagen) following the manufacturer’s protocol. Following proteolytic removal of affinity tags all of the MEKK3 and CCM2 constructs for protein purifications contain vector-derived N-terminal residues with the sequence GS. For mammalian cell expression, EGFP–HA–MEKK3^FL^, EGFP–HA–MEKK3^ΔNPB1^, pcDNA–Flag–CCM2, pmCherry–CCM2 plasmids were constructed by PCR-directed mutagenesis methods using human *MEKK3* and *CCM2* cDNAs^4^,^12^,^13^. EGFP–HA–MEKK3^ΔN^ (1–18 amino-acid deletion), other MEKK3 point mutation expression plasmids (A6D, L7D, A6D/L7D, I10D, L14D, I10D/L14D, A6D/I10D/L14D), and all CCM2 mutation expression plasmids (L299E, M303E, L299E/M303E, A319D, L322D, A319D/L322D) were generated by PCR-directed mutagenesis using PrimeSTAR HS (TaKaRa) following the manufacturer’s protocols. All the mutants were verified by sequencing the whole coding regions in the plasmids.

### Protein expression and purification

The CCM2^HHD^ expression plasmid was transformed into Rosetta (DE3) cells (Novagen) and the MEKK3^NPB1^ expression plasmid into BL21 (DE3) cells (Novagen). After the cells reached *A*_600_=0.6 protein expression was induced by the addition of 0.5 mM isopropyl β-D-1-thiogalactopyranoside (IPTG), and cells were grown at 18 °C overnight. The cells were collected by centrifugation at 2,000 *g* and were resuspended in 500 mM NaCl, 20 mM Tris pH 8.5, DNAseI, dithiothreitol (DTT) and protease inhibitors. The cells were then lysed by three rounds of freeze/thaw in a dry ice/ethanol bath followed by sonication. The lysates were centrifuged at 5,000 *g* for 1 h. Supernatants were filtered and applied to HisTrap columns (GE) and eluted with 500 mM NaCl, 500 mM Imidazole and 20 mM Tris pH 8.5. To produce a CCM2^HHD^:MEKK3^NPB1^ complex 6 × His–CCM2^HHD^ and 6 × His–MEKK3^NPB1^ were then mixed together in a 1:1 molar ratio. TEV protease was added to cleave the 6 × His tags, and the mixture was dialysed overnight against a buffer of 250 mM NaCl, 20 mM Tris pH 8.5. Subsequently, the protein mixture was passed over a second HisTrap column, and the flow through was diluted in 20 mM Tris pH 8.5 to reach a final NaCl concentration of 50 mM. This was then loaded to a Resource Q ion exchange column (GE), and the peak corresponding to the CCM2^HHD^:MEKK3^NPB1^ complex was subjected to a final step of purification by size exclusion chromatography on an S75 column (GE).

### Crystallization

The purified CCM2^HHD^:MEKK3^NPB1^ complex was concentrated to 10 mg ml^*−*1^. Crystallization trials were set-up in 96-well sitting drop plates using a 1:1 protein:precipitant molar ratio (1.0 μl total drop volume). Crystals formed after ∼3 months against a precipitant comprised of 0.2 M potassium phosphate and 20% PEG 3350. The crystals were cryocooled in liquid nitrogen, and data were collected at APS beamline NE-CAT-E at a wavelength of 0.97914 Å and temperature of 100 K.

### Crystal structure determination and refinement

Data were merged and processed using HKL2000 (ref. 29). Molecular replacement was carried out in Phaser^30^ in the CCP4 suite using existing structures of CCM2^HHD^ (PDB ID: 4FQN, chain C) and MEKK3^PB1^ (PDB ID: 2O2V, Chain B) as search models to yield final rotation and translation *Z*-scores of 10.7, 8.9 and 4.2, 4.9 for MEKK3^PB1^ and CCM2^HHD^, respectively. Initial refinement of the solution yielded *R*/*R*_free_=36.4%/39.2%. MEKK3^PB1^ was built into density using phenix.autobuild^31^, and CCM2^HHD^ and MEKK3^N^ were manually built into density using Coot^32^. The structure was refined using phenix.refine^31^ and REFMAC5 (ref. 33) and model building was carried out using Coot. The structure was further refined to final statistics of *R*/*R*_free_=20.8%/23.2%, and has been deposited in the PDB with accession code 4Y5O. All crystallographic software was obtained through the SBGrid consortium^34^. The final Ramachandran plot indicated that 98.3% of residues were in favored regions, while the remaining 1.7% were in allowed regions. The structure has a MolProbity score of 1.61 (99th percentile).

In the crystal structure, we do not observe electron density linking MEKK3^N^ to MEKK3^PB1^ (MEKK3 residues 20–41), however, our SEC-MALS analysis (Supplementary Fig. 9) shows that MEKK3^NPB1^ and CCM2^HHD^ coelute at a stoichiometric 1:1 ratio, and our structure suggests that there is room for the linker between MEKK3^N^ and MEKK3^PB1^ to allow 1:1 packing (distance between carbon alpha atoms of MEKK3 residues 19 and 42 is ∼45 Å, corresponding well to a fully extended loop region of 22 residues at 3.8 Å per residue of ∼84 Å). Therefore, we built MEKK3^N^ to bind the same CCM2^HHD^ molecule as MEKK3^PB1^.

### Pull down assays

GST–MEKK3^NPB1^, GST–MEKK3^N^, GST–MEKK3^PB1^ and GST were transformed into BL21 cells. Overnight cultures were inoculated into LB media and induced with 0.5 mM IPTG when *A*_600_=0.6. Cells were then grown overnight at 18 °C and collected by centrifugation. After resuspending the cell pellets in 1 × PBS supplemented with DTT, lysozyme and protease inhibitor cocktail (Roche), the cells were lysed by three rounds of freeze/thaw followed by sonication. The cell lysates were spun down at 3,500 *g*, and the supernatants were bound to glutathione Sepharose-4B beads (GE) by rotation for 2 h at 4 °C. Beads were washed three times with pull-down buffer (150 mM NaCl, 50 mM Tris pH 7.5, 0.1% Triton-X100) and resuspended to a final protein concentration of 2 mg ml^*−*1^. For the pull-down assay, 5 μl GST-fusion bound bead slurry and 60 μg purified CCM2 in a total 500 μl volume were rotated at 4 °C for 2 h. Samples were washed three times with 1 ml pull-down buffer, run on SDS–polyacrylamide gel electrophoresis (SDS–PAGE), and visualized by staining with Coomassie.

### Coimmunoprecipitation assays

Plasmids encoding HA-tagged MEKK3 or its derived mutants were cotransfected with Flag-tagged CCM2 or its mutants into 293T cells from ATCC. Cells were collected 24 h after transfection and cells were lysed for 30 min on ice in lysis buffer (20 mM Tris-HCl pH 7.4, 250 mM NaCl, 3 mM GDTA, 3 mM EGTA, 0.5% NP-40, 1 mM DTT, 10 mM glycerophosphate, 1 mM sodium vanadate, 0.1% protease inhibitor mixture, 1% PMSF). Flag–CCM2 was immunoprecipitated from the cell lysates with anti-Flag M2 antibody (Sigma) (F1804; dilution 1:1,000). Immunoprecipitates were resolved by an 10% SDS–PAGE electrophoresis, and blotted onto a polyvinylidene difluoride membrane (Millipore). The blotted membranes were blocked with 5% non-fat milk and then incubated with anti-HA from 12CA5 hybridoma^27^ (1:20,000) and anti-Flag (F1804) (1:1,000) antibodies diluted in 5% BSA using a standard immunoblotting procedure and detection by ECL (Millipore).

### Isothermal titration calorimetry

CCM2^HHD^ was purified following a published protocol^13^. Specifically, protein expression was induced when *A*600 ∼0.6 by addition of 1 mM IPTG and shaking at 18 °C overnight. Lysis buffer (250 mM NaCl, 20 mM Tris, 20 mM imidazole, pH 8.5) with DTT, protease inhibitor cocktail (Roche), lysozyme, and DNaseI was used to resuspend the cell pellets, which were then subjected to two rounds of freeze/thaw in dry ice/ethanol and sonicated. The lysate was clarified by centrifugation at 5,000 *g* for 45 min. CCM2^HHD^ was purified by a 1 ml HisTrap HP (GE) column, and TEV protease was used to cleave the 6 × His tag by overnight dialysis at 4 °C. After cleavage, the protein was subjected to a final round of purification by size exclusion chromatography using a Superdex 75 prep grade column (GE).

MEKK3^NPB1^ was purified by nickel affinity chromatography followed by overnight cleavage of the His tag with TEV protease, and a final purification step of size exclusion chromatography. Purified CCM2^HHD^ and MEKK3^NPB1^ were each dialysed into 150 mM NaCl, 50 mM HEPES pH 7.5, filtered through 0.1-μm filters and degassed before ITC experiments. ITC experiments were conducted using a nano-ITC instrument (TA Instruments). CCM2^HHD^ was injected into MEKK3^NPB1^ in 20 2.5-μl injections at 25 °C and 300 r.p.m. The data were processed using the NanoAnalyze software. In the experiments with the mutants (injecting CCM2^HHD^ A319D/A320D mutant into MEKK3^NPB1^ and injecting CCM2^HHD^ into MEKK3^NPB1^ D13R mutant) the same protocol was followed as described above.

### *In vitro* kinase assays

Recombinant full-length Human GST–MEKK3 was purchased from Life Technologies. MKK3 was produced recombinantly as a His-MBP fusion protein. Specifically, Human MKK3 cDNA (Uniprot: P46734) was subcloned into a pMCSG9 vector with an N-terminal HisMBP fusion tag. This was transformed into Rosetta DE3 cells (Novagen). Protein expression was induced by IPTG, and cells were harvested after overnight shaking at 18 °C. The protein was subjected to an initial round of purification by nickel affinity chromatography, followed by size exclusion chromatography using an S200 column (GE). The peak corresponding to HisMBP–MKK3 was collected, concentrated to 100 μM and stored in aliquots at −80 °C.

*In vitro* kinase assays were conducted by mixing 32 nM each of GST–MEKK3, HisMBP-MKK3, and CCM2 in kinase buffer (20 mM Tris pH 7.4, 10 mM MgCl_2_, 1 mM DTT). Kinase reactions were carried out by the addition of 5 mM ATP and incubation at 30 °C for 20 min. Reactions were quenched by the addition of SDS-loading dye and boiling for 5 min. Total CCM2 and total HisMBP–MKK3 were visualized by Coomassie staining, GST–MEKK3 was visualized by western blotting with a GST antibody (AB8902, Millipore) at 1:1,000 dilution, and phosphorylated MKK3 was visualized by western blotting with a phospho-specific antibody for MKK3/6 (Antibody #9231, Cell Signaling Technology) at 1:1,000 dilution. Western blots and Coomassie-stained gels were quantitated using a LiCor Odyssey Imager. Phospho-MKK3 signal for each sample was normalized to the signal from total MKK3, and plotted as fold change in phosphorylation relative to the samples to which CCM2 was not added.

### Isolation of mouse brain microvessel endothelial cells

Primary brain microvessel endothelial cells were isolated according to the procedures modified from the protocol described by Calabria *et al*.^35^. Specifically, 7-day-old brains from the control or *Mekk3 iEC*^*−/−*^ littermate pups were first dissected out carefully. The brain was then rolled gently on a piece of 3-M filter paper three times to remove the big vessels and meninges. Then the white matter was removed from each brain with a surgical blade and all cortices were collected into a sterile Petri dish and cut with scissors into small pieces. Add 10 ml buffer A (NaCl 150 mM, KCL 5.6 mM, CaCl_2_ 2.3 mM, MgCl_2_ 2.6 mM, HEPES 15 mM pH 7.4, BSA 1%) to the tissues and use a 10 ml size pipette to pipet the tissues up and down until no clumps appear. Transfer the suspension to a 50-ml conical tube. Wash Petri dish with 10 ml more buffer A to collect all the tissue. Spin at 270 *g* for 5 min at room temperature on a bench-top centrifuge. Discard the supernatant and suspend the pellet in 2.5 ml 1-mg ml^*−*1^ collagenase, 1 mg ml^*−*1^ dispase in buffer A for 1 h at 37 °C. Spin at 270 *g*, 5 min, resuspend pellets with 5 ml 25% BSA in buffer A. Spin at 1,000 *g* for 20 min at 4 °C. Microvessels will be pelleted at the bottom. The pellets will be further suspended in 1 ml 0.75 mg ml^*−*1^ collagenase, 0.75 mg ml^*−*1^ dispase in buffer A for 20 min at 37 °C, then spin at 270 *g*, 5 min. The pellets were resuspend in 1 ml buffer A and carefully loaded on top of a percoll step gradient (3 ml 25% plus 3 ml 50%) in a 15-ml conical tube. Spin 1,000 *g* for 20 min at 4 °C. Collect the interphase between 25 and 50% percoll. Wash three times with 10 ml buffer A (spin as above). The pellets were resuspended in 2 ml EGM-2 medium (Lonza, New Jersey, USA) as BMEC. Isolated BMEC clusters were seeded on Col IV coated cover slides for 4 days in EGM-2 Medium in the presence of 4 μg ml^*−*1^ puromycin for the first 2 days. *Mekk3* iKO BMECs were either untreated or treated with 1 μM Y27632 for 16 h before being fixed with 3% PFA and 2% Sucrose, followed by staining with anti-VE-Cadherin (Santa Cruz Biotechnology; sc-79818; 1:200) and anti-MLC2pT18/S19 (Cell Signaling Technology; #3674; 1:1,000 for immunoblot, 1:200 for IF). Slides were visualized by a confocol microscope Leica SP8.

### SEC-MALS

The CCM2^HHD^:MEKK3^NPB1^ complex used for crystallization was loaded onto on an Agilent Technologies 1260 HPLC in-line with a DAWN HELEOS II multiangle light-scattering detector (Wyatt Technology) and an Optilab T-rEX differential refractometer (Wyatt Technology). One hundred microliters of a 1.5 mg ml^*−*1^ sample in 150 mM NaCl, 20 mM Tris pH 8.0 and 0.02% sodium azide was run on the system and data were analysed using the Astra6 software (Wyatt Technology).

Full size gels and blots are shown in Supplementary Figs 10, 11 and 12.

## Additional information

**Accession codes:** The structure of MEKK3 with CCM2 is deposited in the Protein Data Bank under accession code 4Y5O.

**How to cite this article:** Fisher, O. S. *et al*. Structure and vascular function of MEKK3–cerebral cavernous malformations 2 complex. *Nat. Commun.* 6:7937 doi: 10.1038/ncomms8937 (2015).

## Supplementary Material

Supplementary Information Supplementary Figures 1-12 and Supplementary Tables 1-4

## Figures and Tables

**Table 1:** Data collection and refinement statistics.

	**CCM2** ^**HHD**^ **:MEKK3** ^**NPB1**^
Data collection	
Space group	*P*2_1_
Cell dimensions	
*a, b, c* (Å)	43.3, 45.4, 61.5
α, β, γ (°)	90, 95.0, 90
Resolution (Å)	50-2.35 (2.43-2.35)*
*R*_sym_ (%)	11.5 (68.8)*
*I*/σ*I*	13.2 (2.5)*
Completeness (%)	97.6 (98.7)*
Redundancy	5.7 (5.5)*
	
Refinement	
Resolution (Å)	36.8-2.35 (2.69-2.35)*
No. of reflections	10,035
*R*_work_/*R*_free_	20.8/23.2 (24.8/32.1)*
No. of atoms	
Protein	1451
Water	20
*B*-factors	
Protein	CCM2: 94.7, MEKK3: 69.3
Water	60.7
R.m.s.d.	
Bond lengths (Å)	0.007
Bond angles (°)	1.018

CCM2, Cerebral cavernous malformations 2; HHD, harmonin homology domain; R.m.s.d, root-mean squared deviations.

One crystal was used to determine the structure.

^*^Highest resolution shell is shown in parentheses.

## References

[b1] Labauge P., Denier C., Bergametti F. & Tournier-Lasserve E. Genetics of cavernous angiomas. Lancet. Neurol. 6, 237–244 (2007). 17303530 10.1016/S1474-4422(07)70053-4

[b2] Cavalcanti D. D. . Cerebral cavernous malformations: from genes to proteins to disease. J. Neurosurg. 116, 122–132 (2012). 21962164 10.3171/2011.8.JNS101241

[b3] Revencu N. & Vikkula M. Cerebral cavernous malformation: new molecular and clinical insights. J. Med. Genet. 43, 716–721 (2006). 16571644 10.1136/jmg.2006.041079 PMC2564569

[b4] Yang J. . Mekk3 is essential for early embryonic cardiovascular development. Nat. Genet. 24, 309–313 (2000). 10700190 10.1038/73550

[b5] Uhlik M. T. . Rac-MEKK3-MKK3 scaffolding for p38 MAPK activation during hyperosmotic shock. Nat. Cell Biol. 5, 1104–1110 (2003). 14634666 10.1038/ncb1071

[b6] Hilder T. L. . Proteomic identification of the cerebral cavernous malformation signaling complex. J. Proteome Res. 6, 4343–4355 (2007). 17900104 10.1021/pr0704276

[b7] Deng Y., Yang J., McCarty M. & Su B. MEKK3 is required for endothelium function but is not essential for tumor growth and angiogenesis. Am. J. Physiol. Cell Physiol. 293, C1404–C1411 (2007). 17687003 10.1152/ajpcell.00058.2007

[b8] Zhou Z. . The cerebral cavernous malformation pathway controls cardiac development via regulation of endocardial MEKK3 signaling and KLF expression. Dev. Cell 32, 168–180 (2015). 25625206 10.1016/j.devcel.2014.12.009 PMC4589864

[b9] Boulday G. . Tissue-specific conditional CCM2 knockout mice establish the essential role of endothelial CCM2 in angiogenesis: implications for human cerebral cavernous malformations. Dis. Model. Mech. 2, 168–177 (2009). 19259391 10.1242/dmm.001263 PMC2650198

[b10] Whitehead K. J. . The cerebral cavernous malformation signaling pathway promotes vascular integrity via Rho GTPases. Nat. Med. 15, 177–184 (2009). 19151728 10.1038/nm.1911 PMC2767168

[b11] Stockton R. A., Shenkar R., Awad I. A. & Ginsberg M. H. Cerebral cavernous malformations proteins inhibit Rho kinase to stabilize vascular integrity. J. Exp. Med. 207, 881–896 (2010). 20308363 10.1084/jem.20091258 PMC2856024

[b12] Fisher O. S. . Structural basis for the disruption of the cerebral cavernous malformations 2 (CCM2) interaction with Krev interaction trapped 1 (KRIT1) by disease-associated mutations. J. Biol. Chem. 290, 2842–2853 (2015). 25525273 10.1074/jbc.M114.616433 PMC4317034

[b13] Fisher O. S. . Structural studies of cerebral cavernous malformations 2 (CCM2) reveal a folded helical domain at its C-terminus. FEBS Lett. 587, 272–277 (2013). 23266514 10.1016/j.febslet.2012.12.011 PMC3558538

[b14] Hu Q. . Insight into the binding properties of MEKK3 PB1 to MEK5 PB1 from its solution structure. Biochemistry 46, 13478–13489 (2007). 17985933 10.1021/bi701341n

[b15] Maddaluno L. . EndMT contributes to the onset and progression of cerebral cavernous malformations. Nature 498, 492–496 (2013). 23748444 10.1038/nature12207

[b16] Liu J. J. . A mechanism of Rap1-induced stabilization of endothelial cell-cell junctions. Mol. Biol. Cell 22, 2509–2519 (2011). 21633110 10.1091/mbc.E11-02-0157 PMC3135476

[b17] Fischer A., Zalvide J., Faurobert E., Albiges-Rizo C. & Tournier-Lasserve E. Cerebral cavernous malformations: from CCM genes to endothelial cell homeostasis. Trends Mol. Med. 19, 302–308 (2013). 23506982 10.1016/j.molmed.2013.02.004

[b18] Borikova A. L. . Rho kinase inhibition rescues the endothelial cell cerebral cavernous malformation phenotype. J. Biol. Chem. 285, 11760–11764 (2010). 20181950 10.1074/jbc.C109.097220 PMC2852911

[b19] Richardson B. T., Dibble C. F., Borikova A. L. & Johnson G. L. Cerebral cavernous malformation is a vascular disease associated with activated RhoA signaling. Biol. Chem. 394, 35–42 (2013). 23096573 10.1515/hsz-2012-0243 PMC3677706

[b20] Wang X., Chang X., Facchinetti V., Zhuang Y. & Su B. MEKK3 is essential for lymphopenia-induced T cell proliferation and survival. J. Immunol. 182, 3597–3608 (2009). 19265138 10.4049/jimmunol.0803738 PMC2923428

[b21] Koni P. A. . Conditional vascular cell adhesion molecule 1 deletion in mice: impaired lymphocyte migration to bone marrow. J. Exp. Med. 193, 741–754 (2001). 11257140 10.1084/jem.193.6.741 PMC2193418

[b22] Zambrowicz B. P. . Disruption of overlapping transcripts in the ROSA beta geo 26 gene trap strain leads to widespread expression of beta-galactosidase in mouse embryos and hematopoietic cells. Proc. Natl Acad. Sci. USA 94, 3789–3794 (1997). 9108056 10.1073/pnas.94.8.3789 PMC20519

[b23] Hayashi S. & McMahon A. P. Efficient recombination in diverse tissues by a tamoxifen-inducible form of Cre: a tool for temporally regulated gene activation/inactivation in the mouse. Dev. Biol. 244, 305–318 (2002). 11944939 10.1006/dbio.2002.0597

[b24] Srinivas S. . Cre reporter strains produced by targeted insertion of EYFP and ECFP into the ROSA26 locus. BMC Dev. Biol. 1, 4 (2001). 11299042 10.1186/1471-213X-1-4 PMC31338

[b25] Wang Y. . Ephrin-B2 controls VEGF-induced angiogenesis and lymphangiogenesis. Nature 465, 483–486 (2010). 20445537 10.1038/nature09002

[b26] Nitta T. . Size-selective loosening of the blood-brain barrier in claudin-5-deficient mice. J. Cell Biol 161, 653–660 (2003). 12743111 10.1083/jcb.200302070 PMC2172943

[b27] Zhang D. . Identification of MEKK2/3 serine phosphorylation site targeted by the Toll-like receptor and stress pathways. EMBO J. 25, 97–107 (2006). 16362041 10.1038/sj.emboj.7600913 PMC1356356

[b28] Li X. . Crystal structure of CCM3, a cerebral cavernous malformation protein critical for vascular integrity. J. Biol. Chem. 285, 24099–24107 (2010). 20489202 10.1074/jbc.M110.128470 PMC2911348

[b29] Otwinowski Z. & Minor W. in Methods in Enzymology Vol. 276, Part A, eds Carter C. W., Sweet R. M. 307–326Academic Press (1997).

[b30] McCoy A. J. Solving structures of protein complexes by molecular replacement with Phaser. Acta. Crystallogr. D Biol. Crystallogr. 63, 32–41 (2007). 17164524 10.1107/S0907444906045975 PMC2483468

[b31] Adams P. D. . PHENIX: a comprehensive Python-based system for macromolecular structure solution. Acta. Crystallogr. D Biol. Crystallogr. 66, 213–221 (2010). 20124702 10.1107/S0907444909052925 PMC2815670

[b32] Emsley P., Lohkamp B., Scott W. G. & Cowtan K. Features and development of Coot. Acta. Crystallogr. D Biol. Crystallogr. 66, 486–501 (2010). 20383002 10.1107/S0907444910007493 PMC2852313

[b33] Murshudov G. N. . REFMAC5 for the refinement of macromolecular crystal structures. Acta. Crystallogr. D Biol. Crystallogr. 67, 355–367 (2011). 21460454 10.1107/S0907444911001314 PMC3069751

[b34] Morin A. . Cutting edge: Collaboration gets the most out of software. Elife 2, e01456 (2013). 24040512 10.7554/eLife.01456 PMC3771563

[b35] Calabria A. R., Weidenfeller C., Jones A. R., de Vries H. E. & Shusta E. V. Puromycin-purified rat brain microvascular endothelial cell cultures exhibit improved barrier properties in response to glucocorticoid induction. J. Neurochem. 97, 922–933 (2006). 16573646 10.1111/j.1471-4159.2006.03793.x

[b36] McNicholas S., Potterton E., Wilson K. S. & Noble M. E. Presenting your structures: the CCP4mg molecular-graphics software. Acta. Crystallogr. D Biol. Crystallogr. 67, 386–394 (2011). 21460457 10.1107/S0907444911007281 PMC3069754

[b37] Laskowski R. A. PDBsum new things. Nucleic. Acids. Res. 37, D355–D359 (2009). 18996896 10.1093/nar/gkn860 PMC2686501

[b38] Kim K., Duramad O., Qin X. F. & Su B. MEKK3 is essential for lipopolysaccharide-induced interleukin-6 and granulocyte-macrophage colony-stimulating factor production in macrophages. Immunology 120, 242–250 (2007). 17116170 10.1111/j.1365-2567.2006.02495.x PMC2265862

