# Supplementary material for: Structure and vascular function of MEKK3–cerebral cavernous malformations 2 complex
Source: Nat Commun. 2015 Aug 3;6:7937. doi: 10.1038/ncomms8937 (PMC4526114; doi:10.1038/ncomms8937)
Supplement: Supplementary Information — Supplementary Figures 1-12 and Supplementary Tables 1-4 [file ncomms8937-s1.pdf]

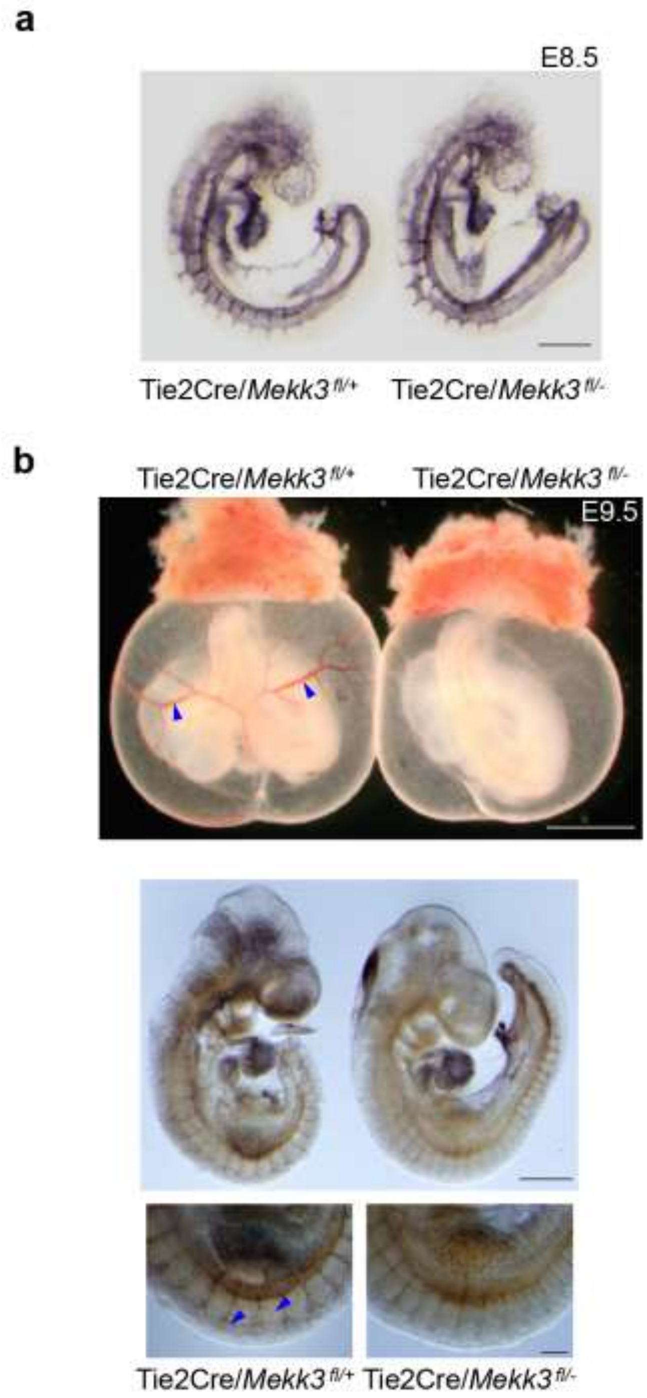

Supplementary Figure 1. ***Mekk3*** **plays an endothelial-specific role in development.**

(a) E8.5 *Mekk3* NCL (Tie2Cre/*Mekk3*$^{fl/+}$) and *Mekk3* EC-cKO (Tie2Cre/*Mekk3*$^{fl/-}$) embryos were whole mount stained for CD31. Scale bar indicates 300 µm. **(b)** E9.5 *Mekk3* NCL and *Mekk3* EC-cKO embryos were visualized by a dark field microscope (top panels), or were whole-mount

stained for CD31 (middle and bottom panels). The vasculatures in somatic areas were shown at a higher magnified view (bottom panels). Arrowheads show extensive angiogenesis in the NCL embryo but not the *Mekk3* EC-cKO embryo. Top scale bar indicates 600 µm, middle indicates 500 µm, bottom indicates 100 µm.

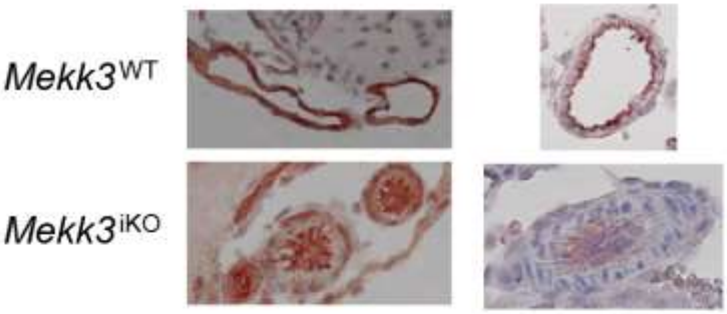

Supplementary Figure 2. *Mekk3* maintains neonatal brain vasculature patency.

Mekk3 WT and *Mekk3* iKO brain sections were stained for CD31 (brown) as indicated. Power of objective lens: 63X.

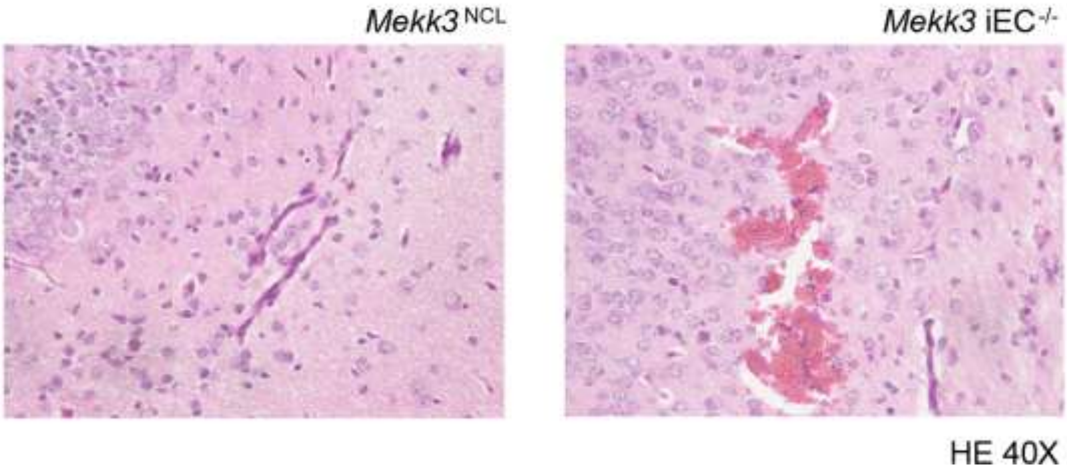

Supplementary Figure 3. High power magnification of H&E staining of neonatal brain sections. Hematoxylin and eosin staining of paraffin-embedded sections of *Mekk3*^{NCL} and *Mekk3* iEC$^{-/-}$ brains.

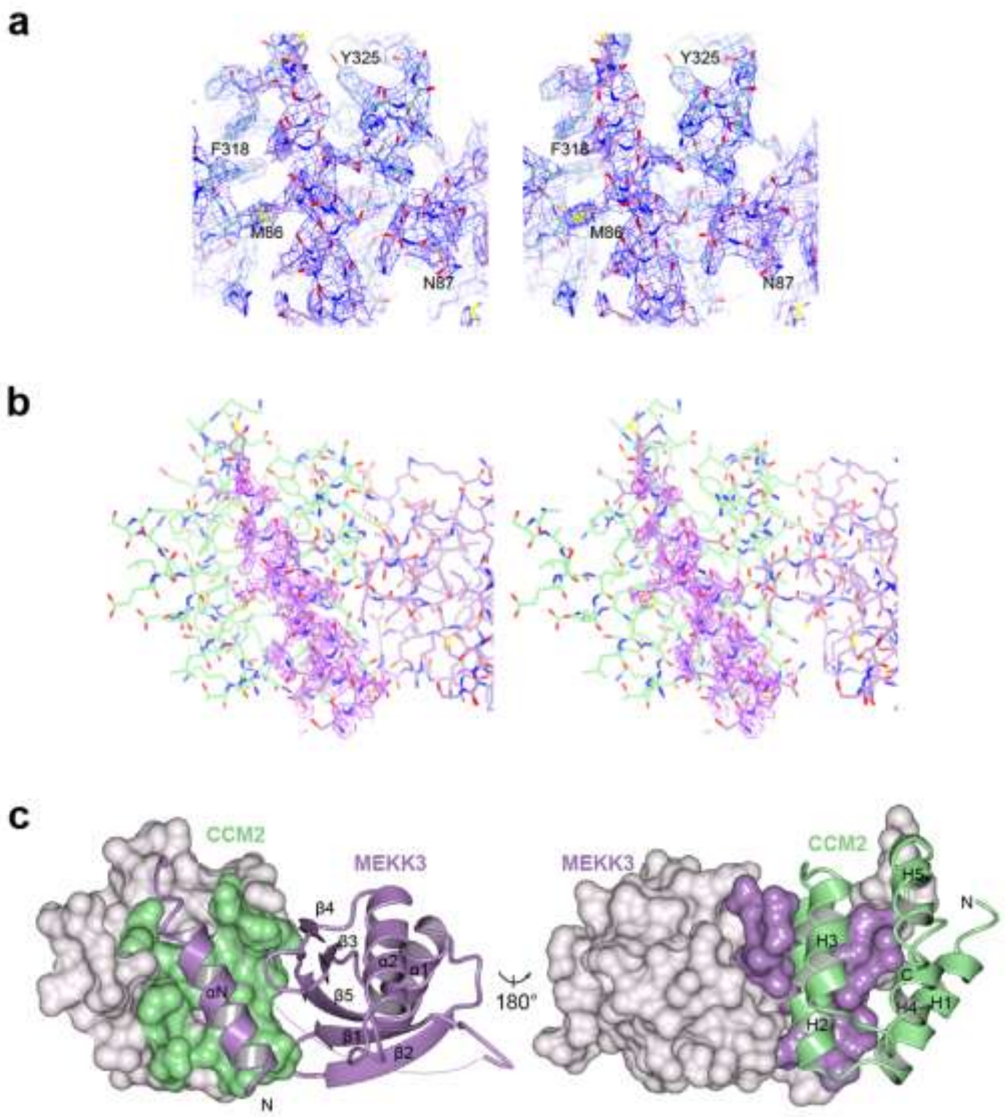

Supplementary Figure 4. Structure of CCM2^{HHD}: MEKK3^{NPB1} complex.

(a) Stereoview showing representative 2F_{obs}-F_{calc} electron density map contoured at 1.5 σ. CCM2^{HHD} is shown as green sticks and MEKK3^{NPB1} as purple sticks. **(b)** Stereoview showing simulated annealing omit map (purple) for MEKK3 αN contoured at 3σ. The final refined model is shown in stick format. For clarity the map is clipped at 1.5 Å away from MEKK3 αN. **(c)** Left panel shows CCM2 in surface representation with residues involved in the MEKK3 interaction in

green, and MEKK3 represented as ribbons. Right panel shows MEKK3 as a surface with CCM2 interacting residues highlighted in purple with CCM2 depicted as ribbons. The amino acid sequences of proteins crystallized were MEKK3: GSMDEQEALNSIMNDLVAL QMNRRHRMPGYETMKNKDTGHSNRQSDVRIKFEHNGERRIIAFSRPVKYEDVEHKVTTVFG QPLDLHYMNNELSILLKNQDDLDKAIDILDRSSSMKSLRILLLSQD and CCM2: GSKTISESELS ASATELLQDYMLTLRTKLSSQEIQQFAALLHEYRNGASIHEFCINLRQLYGDSRKFLLLGLRPF IPEKDSQHFENFLETIGVKDGRG.

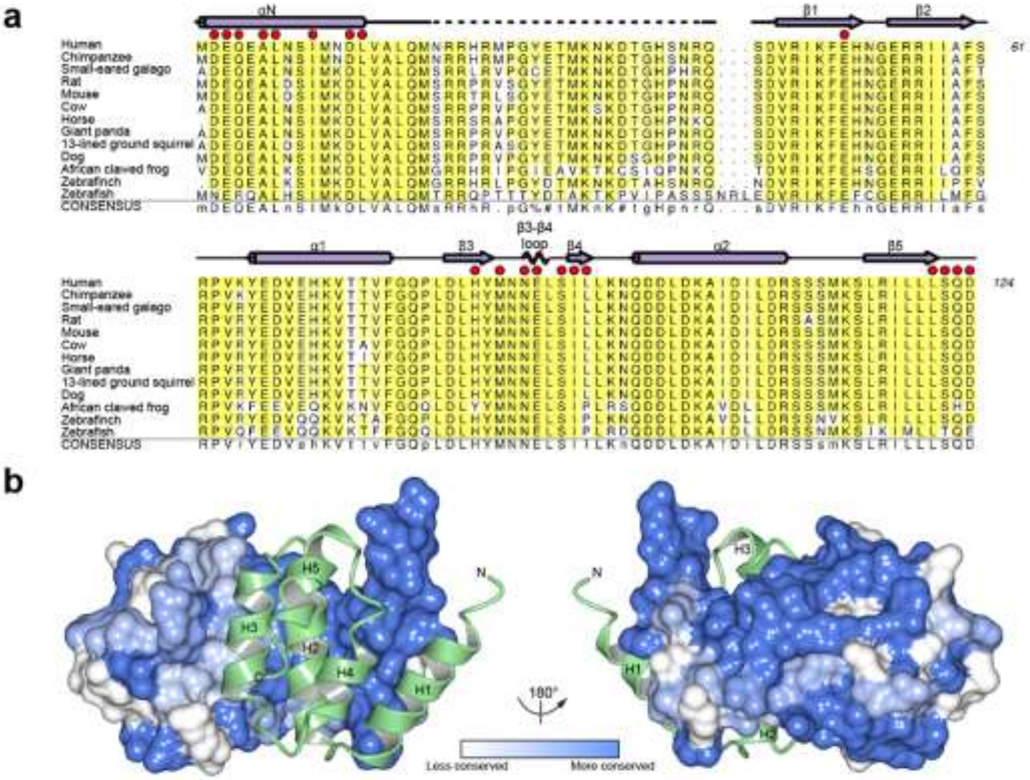

Supplementary Figure 5. MEKK3 alignment and interacting residues.

(a) Sequence conservation of MEKK3^{NPB1} over evolution. Crystallographically-determined secondary structure elements are indicated above the alignment with α-helices shown as cylinders, β-strands as arrows, 3_{10} helix as a wave, and unstructured residues as a dashed line. Residues involved in the interaction with CCM2 are denoted by red dots. Species shown are: Q99759, *Homo sapiens* (indicated as Human); K6ZLE7, *Pan troglodytes* (Chimpanzee); H0WV28, *Otolemur garnettii* (Small-eared galago); B5DF98, *Rattus norvegicus* (Rat); Q61084, *Mus musculus* (Mouse); F1MH06, *Bos taurus* (Cow); F6WM48, *Equus caballus* (Horse); G1L9C7, *Ailuropoda melanoleuca* (Giant panda); I3MCG3, *Spermophilus tridecemlineatus* (13-lined ground squirrel); F1P750, *Canis familiaris* (Dog); F6X5R9, *Xenopus tropicalis* (African

clawed frog); H0YUP8, *Taeniopygia guttata* (Zebrafinch); and E7EXX1, *Danio rerio* (Zebrafish).

(b) Surface conservation of MEKK3. Completely conserved residues are shown in blue. Conservation over 13 sequences and color coding calculated using the Consurf server[37]. MEKK3 shown as a surface, CCM2 is shown in cartoon format and colored green.

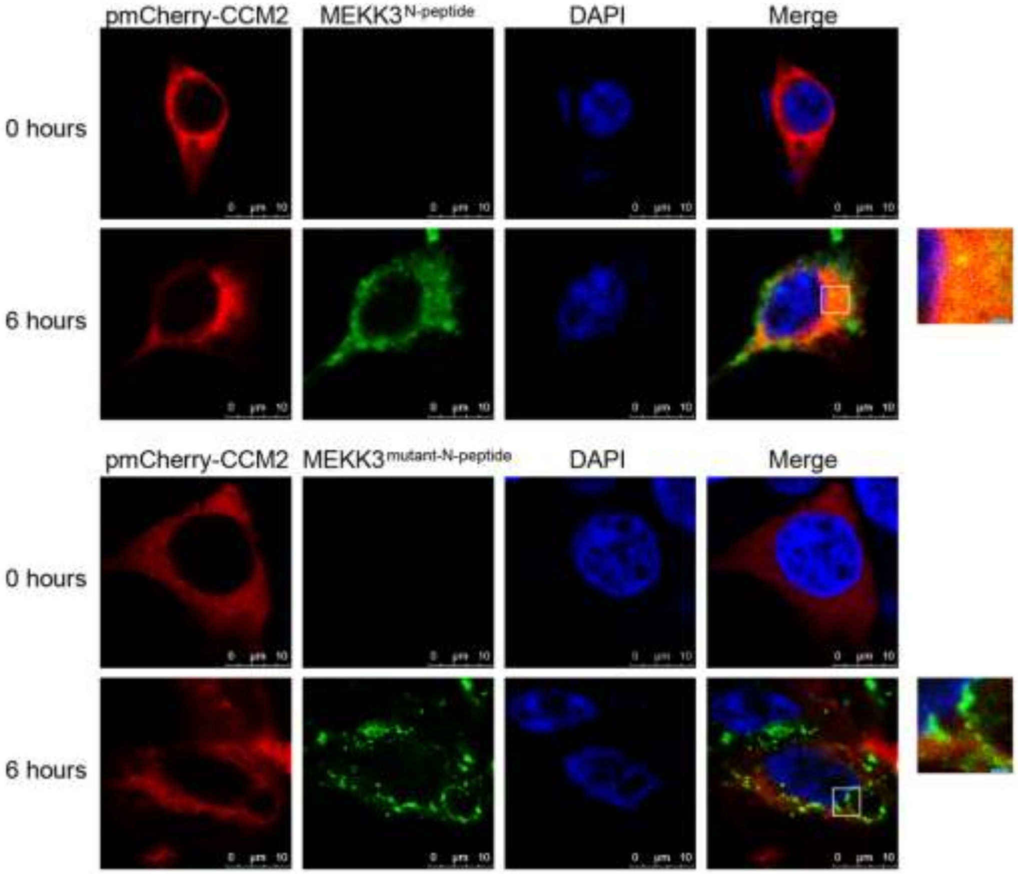

Supplementary Figure 6. Visualization of cell permeable peptides in cultured cells.

HeLa cells transfected with pmCherry-CCM2 were incubated with MEKK3$^{N\text{-}peptide}$ or MEKK3$^{mutant\text{-}N\text{-}peptide}$ for at 0 or 6 h. Cells were stained with DAPI for nucleus (Blue), and pmCherry-CCM2 and peptides were visualized with a Leica LM8 confocal microscopy. White scale bars indicate 10 µm; cyan scale bars indicate 1 µm.Power of objective lens: 63X.

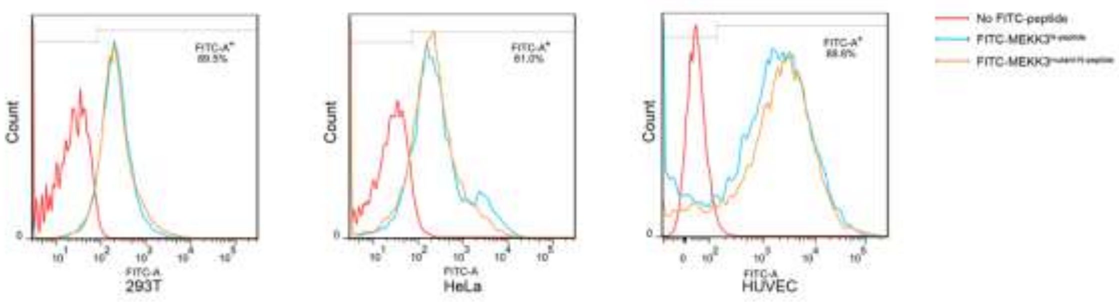

Supplementary Figure 7. Cells loaded with cell permeable peptides assayed by flow cytometry. HeLa or HUVEC cells were incubated with MEKK3$^{N\text{-}peptide}$ or MEKK3$^{mutant\text{-}N\text{-}peptide}$ for 24 h and the cells were trypsinized and analyzed by flow cytometry for peptide-loaded cells.

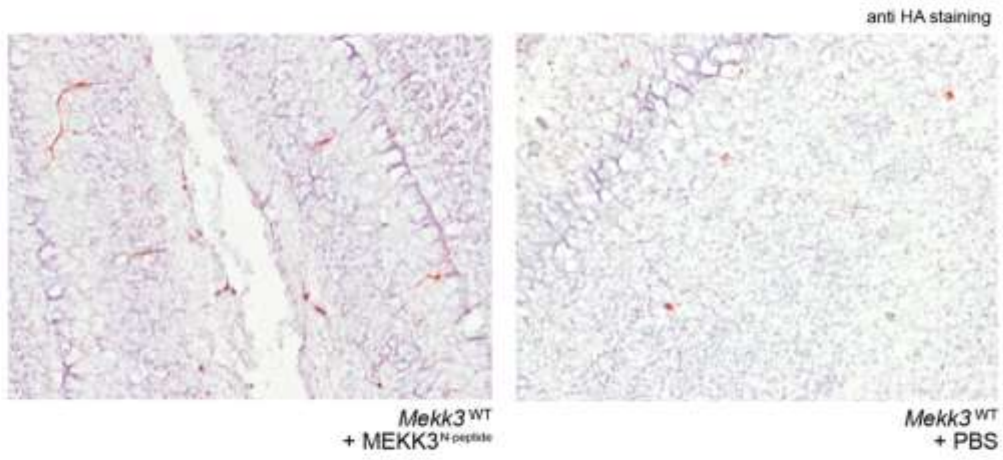

Supplementary Figure 8. Staining for cell permeable peptides in brain sections.

Paraffin-embedded brain sections of *Mekk3*^{WT} pups treated with HA-tagged cell permeable peptides were immunostained with an anti-HA antibody. Power of objective lens: 10X.

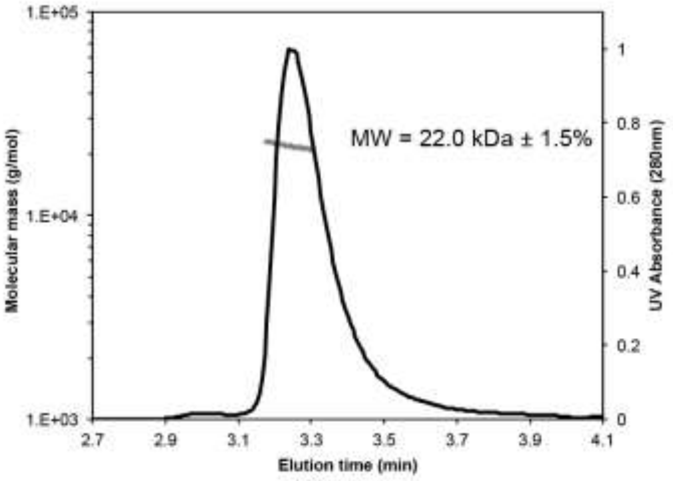

Supplementary Figure 9. SEC-MALS shows a 1:1 complex of MEKK3^{NPB1} and CCM2^{HHD}. Size exclusion chromatography with Multi-Angle Light Scattering for MEKK3^{NPB1}:CCM2^{HHD} complex indicates a molecular mass for the complex of 22 kDa ± 1.5%, compared to the expected molecular mass for a 1:1 complex of 25.5 kDa.

Fig 1i

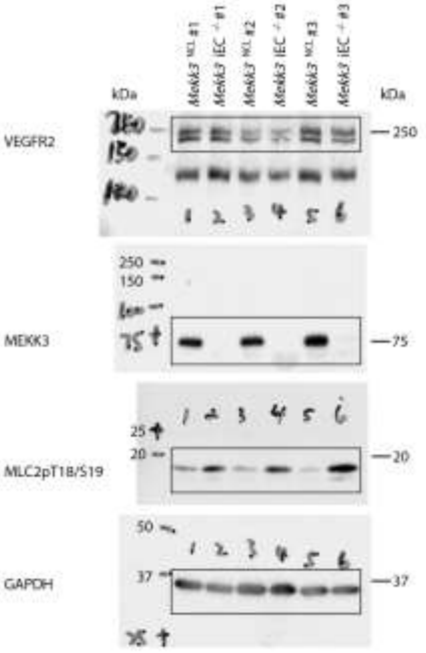

Fig 2a

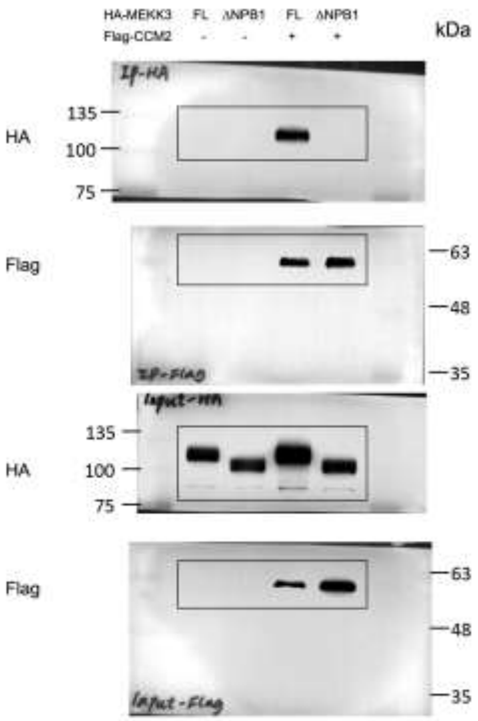

Fig 2c

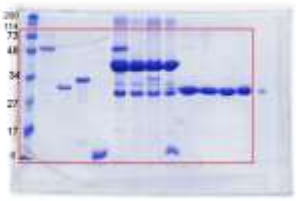

Fig 2d

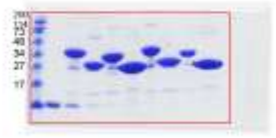

Fig 2h

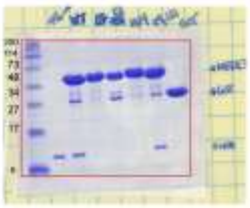

Fig 2i

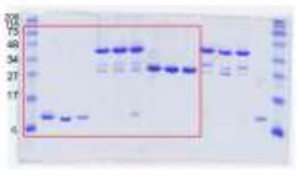

Supplementary Figure 10. Full size gels and blots.

Fig 3a

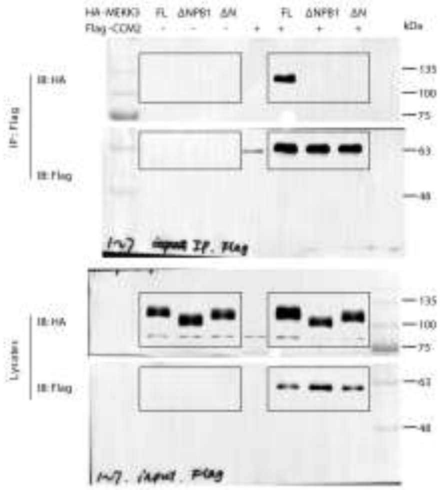

Fig 3b

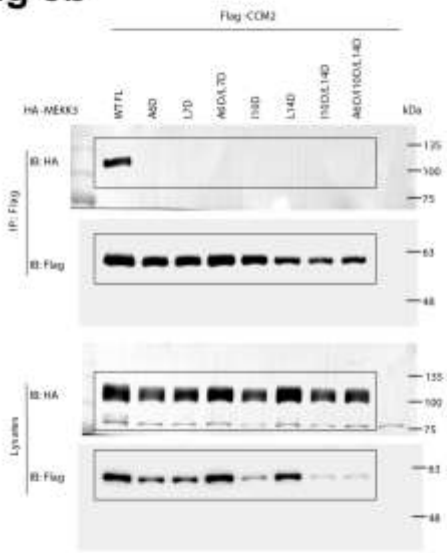

Fig 3c

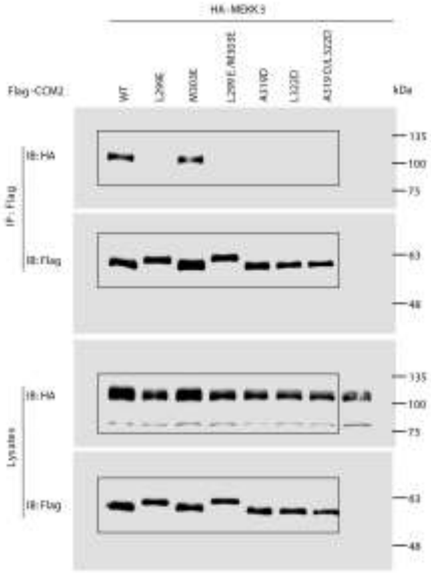

Fig 3d

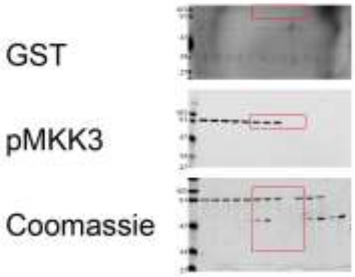

Supplementary Figure 11. Full size gels and blots.

Fig 3e

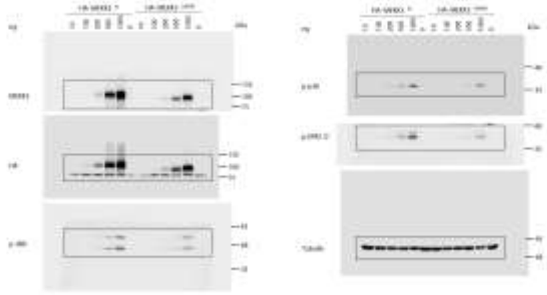

Fig 3f

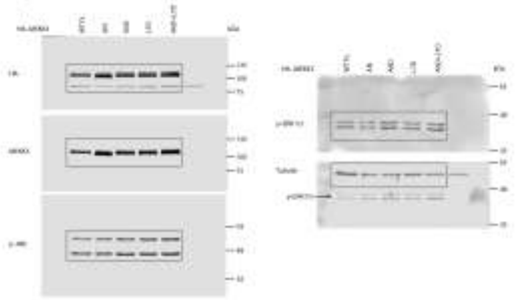

Fig 3h

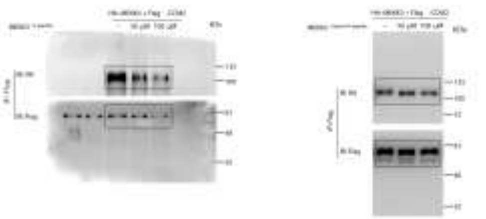

Fig 3i

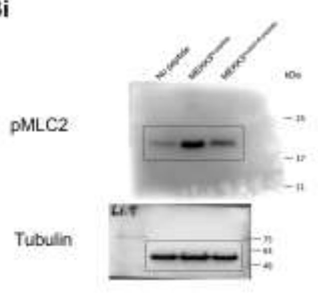

Supplementary Figure 12. Full size gels and blots.

Supplementary Table 1. Isothermal Titration Calorimetry measurements. NB indicates no binding observed.

CCM2^{HHD} (titrant)	MEKK3^{NPB1} (sample cell)	K_d (μM)	N (stoichiometry)	ΔH (kJ/mol)	ΔS (J/mol*K)	K_a (1/M)
550 μM WT	39 μM WT	1.23	0.90	-17.1	55.4	789000
620 μM WT	86 μM WT	1.02	0.46	-8.3	86.7	976000
660 μM WT	96 μM WT	0.79	0.29	-9.4	85.3	1260000
633 μM WT	135 μM WT	2.59	0.53	-14.5	58.3	386000
**Average**		1.41	0.54	-12.3	71.4	852750
**Standard Deviation**		0.81	0.26	4.2	16.9	366498
**Standard Error of the Mean**		0.36	0.11	1.9	7.6	163903
600 μM WT	80 μM D13R	NB				
600 μM WT	80 μM A6D/L7D	NB				
620 μM A319D/A320D	86 μM WT	NB				

Supplementary Table 2. 7 Day survival table for different doses of Y27632. Number of surviving pups indicated.

Rescued with 10 µg/g Y27632, fed starting Day 1							
Days	1	2	3	4	5	6	7
WT	4	4	4	4	4	4	4
EC-iKO	5	5	5	3	3	2	0
Rescued with 2.5 µg/g Y27632							
Days	1	2	3	4	5	6	7
WT	4	4	4	4	4	4	4
EC-iKO	2	2	2	2	2	1	0
Rescued with 2 µg/g Y27632							
Days	1	2	3	4	5	6	7
WT	5	5	5	5	5	5	5
EC-iKO	2	2	2	1	1	1	1
Rescued with 1 µg/g Y27632							
Days	1	2	3	4	5	6	7
WT	3	3	3	3	3	3	3
EC-iKO	5	5	5	5	5	5	5

Supplementary Table 3. Survival data on treatment with Y27632. Data used to generate Figure 4e. Number of surviving pups indicated.

Days	1	2	3	4	5	6	7	8	9	10	11	12	13	14	15	16	17	18	19	20
Mekk3 NCL (number of mice surviving)	16	16	16	16	16	16	16	16	16	16	16	16	16	16	16	16	16	16	16	16
Mekk3-iEC-/- (number of mice surviving)	16	16	16	15	15	13	11	9	7	7	7	7	7	6	6	6	6	5	5	5
Mekk3 NCL +Y27632 (number of mice surviving)	16	16	16	16	16	16	16	16	16	16	13	13	13	13	13	13	13	13	13	13
Mekk3-iEC-/- +Y27632 (number of mice surviving)	13	13	13	13	13	13	12	12	10	9	9	9	8	7	6	5	5	5	5	5

Supplementary Table 4. Primers used.

MEKK3 ΔN	Forward	CGACGTGCCGGACTACGCTTCCatgagccgacgaacccggttgtctgg
	Reverse	ccagacaaccgggttcgtcggctcatGGAAGCGTAGTCCGGCACGTCG
MEKK3 A6D	Forward	gatgaacaagagGATttagactcgatcatgaaggacctgg
	Reverse	ccaggtccttcatgatcgagtctaaATCctcttgttcatc
MEKK3 L7D	Forward	gatgaacaagaggcaGATgactcgatcatgaaggacctgg
	Reverse	ccaggtccttcatgatcgagtcATCtgcctcttgttcatc
MEKK3 A6D/L7D	Forward	atggatgaacaagagGATGATgactcgatcatgaaggacctggtg
	Reverse	caccaggtccttcatgatcgagtcATCATCctcttgttcatccat
MEKK3 I10D	Forward	caagaggcattagactcgGACatgaaggacctggtggccc
	Reverse	gggccaccaggtccttcatGTCcgagtctaatgcctcttg
MEKK3 L14D	Forward	gactcgatcatgaaggacGATgtggccctccagatgagccg
	Reverse	cggctcatctggagggccacATCgtccttcatgatcgagtc
MEKK3 I10D/L14D	Forward	gaacaagaggcattagactcgGACatgaaggacGATgtggccctccagatgagccgacg
	Reverse	cgtcggctcatctggagggccacATCgtccttcatGTCcgagtctaatgcctcttgttc
MEKK3 A6D/I10D/L14D	Forward	gaacaagagGATttagactcgGACatgaaggacGATgtggccctccagatgagccgacg
	Reverse	cgtcggctcatctggagggccacATCgtccttcatGTCcgagtctaaATCctcttgttc
MEKK3 D13R	Forward	gcattagactcgatcatgaagAGGctggtggccctccagatgag
	Reverse	ctcatctggagggccaccagCCTcttcatgatcgagtctaatgc
CCM2 L299E	Forward	gccagcgccactgagctgGAGcaggactacatgctgacgc
	Reverse	gcgtcagcatgtagtcctgCTCcagctcagtggcgctggc
CCM2 M303E	Forward	gagctgctgcaggactacGAGctgacgctgcgcaccaagc
	Reverse	gcttggtgcgcagcgtcagCTCgtagtcctgcagcagctc
CCM2 L299E/M303E	Forward	gcgccagcgccactgagctgGAGcaggactacGAGctgacgctgcgcaccaagc
	Reverse	gcttggtgcgcagcgtcagCTCgtagtcctgCTCcagctcagtggcgctggcgc
CCM2 A319D	Forward	cacaggagatccagcagtttGATgcactgctgcacgagtaccgc
	Reverse	gcggtactcgtgcagcagtgcATCaaactgctggatctcctgtg
CCM2 L322D	Forward	ccagcagtttgcagcactgGACcacgagtaccgcaatggg
	Reverse	cccattgcggtactcgtgGTCcagtgctgcaaactgctgg
CCM2 A319D/L322D	Forward	catcacaggagatccagcagtttGATgcactgGACcacgagtaccgcaatggggcc
	Reverse	ggccccattgcggtactcgtgGTCcagtgcATCaaactgctggatctcctgtgatg
CCM2 A320D	Forward	cacaggagatccagcagtttgcaGATctgctgcacgagtaccgc
	Reverse	gcggtactcgtgcagcagATCtgcaaactgctggatctcctgtg
CCM2 A319D/A320D	Forward	cacaggagatccagcagtttGATGATctgctgcacgagtaccgcaatggg
	Reverse	cccattgcggtactcgtgcagcagATCATCaaactgctggatctcctgtg
GST-MEKK3 1-124	Forward	atc ta**g aat tc**g atg gac gaa cag gag gca
	Reverse	G GGG TCG AC A TCA GTC CTG GGA CAA CAG CAA
GST-MEKK3 1-36	Forward	gaccatgaagaacaaagacacatagcactcaaataggcagagtgacg
	Reverse	cgtcactctgcctatttgagtgctatgtgtctttgttcttcatggtc

GST-MEKK3 37-124	Forward	atc ta**g aat tc**g ggt cac tca aat agg cag
	Reverse	G GGG TCG AC A TCA GTC CTG GGA CAA CAG CAA
His-CCM2 1-438	Forward	atcggatccatggaagaggagggcaagaag
	Reverse	ccggaattcattagctgcagcccagcgcctcaat
His-CCM2 51-251	Forward	atcggatccgagcgcgtcgagccagacaga
	Reverse	ccggaattcattaagagtcatcgctgtgcaggga
His-CCM2 231-438	Forward	ATC GGATCC AGA GCG ATA TTT GAT GGG GCC TCT
	Reverse	ccggaattcattagctgcagcccagcgcctcaat
His-CCM2 283-379	Forward	gaaggatggccgcggctagatcactgacagctttgg
	Reverse	ccaaagctgtcagtgatctagccgcggccatccttc
CCM2 A319D/A320D	Forward	caggagatccagcagtttgatgatctgctgcacgagtaccgca
	Reverse	tgcggtactcgtgcagcagatcatcaaactgctggatctcctg
CCM2 A319D/A322D	Forward	caggagatccagcagtttgatgcactggatcacgagtaccgcaatgggg
	Reverse	ccccattgcggtactcgtgatccagtgcatcaaactgctggatctcctg
MEKK3 A6D/L7D	Forward	gggaattcgatggacgaacaggaggatgataactcaatcatgaacgatctggtgg
	Reverse	ccaccagatcgttcatgattgagttatcatcctcctgttcgtccatcgaattccc
MEKK3 D13R	Forward	cattgaactcaatcatgaaccgtctggtggccctccagatg
	Reverse	catctggagggccaccagacggttcatgattgagttcaatg
MEKK3 I10D/L14D	Forward	acaggaggcattgaactcagacatgaacgatgatgtggccctccagatgaacc
	Reverse	ggttcatctggagggccacatcatcgttcatgtctgagttcaatgcctcctgt
MEKK3 L119E/L121E	Forward	agaagctcaagcatgaaaagccttaggatagagctggagtcccaggactgatgt
	Reverse	acatcagtcctgggactccagctctatcctaaggcttttcatgcttgagcttct
For genotyping Mekk3 KO	#105	TCG CAG CGC ATC GCC TTC TA
For genotyping Mekk3 KO	#107	ATG TGA AGC TTG GGG ATT TTG
For genotyping Mekk3 KO	#174	TGG TTA GAC TCA CTG GTC AGA GAC
Mekk3+		#107 and #174 (900 bp)
Mekk3-		#107 and #105 (830 bp)
Mekk3 Flox		#107 and #174 (1000 bp)
